# A comprehensive approach to developing a multi-epitope vaccine against *Mycobacterium tuberculosis*: from *in silico* design to *in vitro* immunization evaluation

**DOI:** 10.3389/fimmu.2023.1280299

**Published:** 2023-11-02

**Authors:** Fan Jiang, Yong Han, Yinping Liu, Yong Xue, Peng Cheng, Li Xiao, Wenping Gong

**Affiliations:** ^1^ Beijing Key Laboratory of New Techniques of Tuberculosis Diagnosis and Treatment, Senior Department of Tuberculosis, The Eighth Medical Center of PLA General Hospital, Beijing, China; ^2^ Respiratory Research Institute, Senior Department of Pulmonary & Critical Care Medicine, The Eighth Medical Center of PLA General Hospital, Beijing, China; ^3^ Section of Health, No. 94804 Unit of the Chinese People’s Liberation Army, Shanghai, China; ^4^ Resident standardization training cadet corps, Air Force Hospital of Eastern Theater, Nanjing, China

**Keywords:** *Mycobacterium tuberculosis* (MTB), tuberculosis (TB), multi-epitope vaccine (MEV), immunoinformatic, enzyme linked immunospot assay (ELISPOT), cytometric bead assay (CBA)

## Abstract

**Introduction:**

The Bacillus Calmette-Guérin (BCG) vaccine, currently used against tuberculosis (TB), exhibits inconsistent efficacy, highlighting the need for more potent TB vaccines.

**Materials and methods:**

In this study, we employed reverse vaccinology techniques to develop a promising multi-epitope vaccine (MEV) candidate, called PP13138R, for TB prevention. PP13138R comprises 34 epitopes, including B-cell, cytotoxic T lymphocyte, and helper T lymphocyte epitopes. Using bioinformatics and immunoinformatics tools, we assessed the physicochemical properties, structural features, and immunological characteristics of PP13138R.

**Results:**

The vaccine candidate demonstrated excellent antigenicity, immunogenicity, and solubility without any signs of toxicity or sensitization. *In silico* analyses revealed that PP13138R interacts strongly with Toll-like receptor 2 and 4, stimulating innate and adaptive immune cells to produce abundant antigen-specific antibodies and cytokines. *In vitro* experiments further supported the efficacy of PP13138R by significantly increasing the population of IFN-γ^+^ T lymphocytes and the production of IFN-γ, TNF-α, IL-6, and IL-10 cytokines in active tuberculosis patients, latent tuberculosis infection individuals, and healthy controls, revealing the immunological characteristics and compare the immune responses elicited by the PP13138R vaccine across different stages of *Mycobacterium tuberculosis* infection.

**Conclusion:**

These findings highlight the potential of PP13138R as a promising MEV candidate, characterized by favorable antigenicity, immunogenicity, and solubility, without any toxicity or sensitization.

## 1 Introduction

*Mycobacterium tuberculosis* (MTB) is an acid-fast intracellular bacterium that requires oxygen for survival. Although MTB has been recognized for millennia, it was not until 1882 that Robert Koch demonstrated its role as the causative agent of human tuberculosis (TB) (1). While TB can affect any organ or tissue in the body except hair and nails, pulmonary tuberculosis (PTB) is the most common form of the disease. Unfortunately, TB remains a significant public health challenge, with 98% of cases occurring in low- and middle-income countries (LMICs) (2–5). The World Health Organization (WHO) reported 10.6 million new TB cases and 1.6 million deaths worldwide in 2021 (6). Disturbingly, the COVID-19 pandemic has further exacerbated the challenges facing TB control, with a 4.5% increase in global incidence (from 10.1 million in 2020) and a rise in TB mortality (1.5 million in 2020; 1.4 million in 2019) following years of progress in reducing TB burden (6).

Vaccination is the most effective and cost-efficient method for preventing and controlling infectious diseases (7). Notably, the Bacillus Calmette-Guérin (BCG) vaccine, developed nearly 100 years ago using an attenuated strain of *M. bovis*, remains the only TB vaccine available. While BCG vaccination affords high protection against meningeal and miliary TB in infants and children, it confers inadequate protection (0-80%) against PTB, particularly in adults (8, 9). Consequently, there exists a pressing need to develop improved TB vaccines. Several TB vaccines are currently in various stages of clinical development, including MIP, VPM1002, MTBVAC, GamTBvac, BCG (re)vaccination, M72/AS01E, DAR-901, H56:IC31, ID93/GLA-SE, RUTI, ChAdOx1.85A +MVA85A, and AEC/BC02, BNT164, TB/FLU-05E, TB/FLU-01L, TB/FLU-04L, and AdHu5Ag85A. Among these, the M72/AS01E vaccine stands out as a promising candidate (10). The M72/AS01E vaccine is a subunit vaccine that combines the fusion protein M72 and the adjuvant AS01E. The M72 protein is derived from two MTB antigens, Mtb32A and Mtb39A, both known to elicit robust immune responses (11–13). However, the M72/AS01E subunit vaccine lacks in-depth analysis of CTL, HTL, and B cell epitopes, and the extent to which it can effectively activate CD4^+^, CD8^+^ T lymphocytes, and B lymphocytes remains unconfirmed. This might explain why the final protective efficacy of the vaccine was only 49.7% at month 36, falling slightly below the minimum requirement set by the WHO (14).

Several studies have provided evidence indicating that the efficient stimulation of CD4^+^, CD8^+^ T lymphocytes, and B lymphocytes, leading to the generation of immune responses, is pivotal for subunit vaccine effectiveness (3, 15–20). Nevertheless, conventional approaches to subunit vaccine development depend on one or more antigens, lacking the ability to forecast and evaluate the prevailing epitopes for THL, CTL, and B lymphocytes on each antigen. This limitation not only diminishes the vaccine’s efficacy but also elevates the likelihood of adverse reactions (21). Fortunately, the rise of bioinformatics and immunoinformatics has brought a ray of light to a dark time in TB subunit vaccine research. Bioinformatics-based antigenic epitope prediction, epitope screening, linker selection, and intramolecular adjuvant use are unique features of multi-epitope vaccines (MEVs), which are considered to be an effective method to reduce costs and save time, allowing the development of MEVs with maximum protective efficacy and minimal adverse effects (22, 23). Furthermore, immunoinformatics can be used at the vaccine design stage to evaluate the immunological properties of MEVs, such as their ability to induce the proliferation of innate and adaptive immune cells, their capacity to stimulate immune cells to produce key cytokines such as IFN-γ, IL-4 and IL-10, and their sensitization and toxicity (24–26). Given the successful development of bioinformatics and immunoinformatics technology in reverse vaccinology, on March 10, 2023, the journal *FEMS Microbiology Reviews* published A guide to current methodology and usage of reverse vaccinology towards in silico vaccine discovery (27). This is the first comprehensive introduction to the concept, development, and mainstream tools of bioinformatics and immunoinformatics technology, which will provide valuable guidance to all researchers wishing to apply this new technology to MEV design.

TB vaccine research has also experienced the invigorating influence of the rapid progress in bioinformatics and immunoinformatics (10). Particularly, since 2020, there has been a surge in studies focusing on TB MEVs (3, 28–34). In our previous study, we reported the method of correctly using bioinformatics and immunoinformatics tools to design TB MEVs and developed several novel MEVs based on these tools (15, 17, 18, 20, 35, 36). This investigation successfully developed a novel MEV targeting TB by incorporating 13 helper T lymphocytes (HTL) epitopes, 13 cytotoxic T lymphocytes (CTL) epitopes, and 8 B-cell epitopes derived from 17 potential antigens of MTB. The immunogenicity, antigenicity, cytokine-inducing capacity, sensitization potential, and toxicity characteristics of this MEV were thoroughly analyzed utilizing bioinformatics and immunoinformatics tools. In comparison to previously reported TB MEVs, our MEV demonstrates several notable advantages, including a wider range of epitopes, heightened reliability, enhanced immunogenicity, and reduced side effects. Additionally, we assessed the population coverage of the MEV across human leukocyte antigen I (HLA-I) and HLA-II alleles, as well as conducted *in silico* evaluation of its immunological profile. Importantly, these predicted immunological profiles were further validated through *in vitro* experiments. This study not only introduces innovative strategies for designing novel TB vaccines but also identifies potential targets for developing a new generation of safer and more efficacious TB MEVs.

## 2 Materials and methods

### 2.1 Experimental design and medical ethics

In this investigation, we utilized *in silico* tools to generate the innovative MEV, which was subsequently subjected to immunological assessment using peripheral blood mononuclear cells (PBMCs) from patients with active tuberculosis (ATB), individuals with latent tuberculosis infection (LTBI) and healthy controls (HCs). The study was conducted between April 2022 and December 2022 at the Senior Department of Tuberculosis, the Eighth Medical Center of PLA General Hospital. Ethical approval for experiments involving PBMCs from HCs, patients with ATB, and individuals with LTBI was granted by the Ethics Committee of the Eighth Medical Center of PLA General Hospital (approval number: 309202204080808). All participants provided informed consent before being enrolled in the study.

### 2.2 Inclusion and exclusion criteria of subjects

The selection criteria for HCs, ATB patients, and individuals with LTBI adhered to the TB diagnostic criteria (WS288-2017) established by the National Health and Family Planning Commission of China (NHFPC). All the volunteers recruited for this study were vaccinated with BCG at birth. Briefly, HCs were included if they met the following criteria: 1) no prior exposure to ATB patients; 2) negative interferon-gamma release test (IGRA) or EC test [recombinant MTB fusion protein of early secretory antigenic target protein 6-kDa (ESAT-6) and culture filtrate protein 10 kDa (CFP-10)]; 3) absence of ATB clinical symptoms; 4) negative for HIV (human immunodeficiency virus); and 5) normal chest X-ray results indicating no diagnosis of ATB. Conversely, exclusion criteria for HCs were as follows: 1) history of residing or traveling in a high-risk TB area, 2) history of working in a TB laboratory or hospital, 3) age below 12 years, 4) history of TB or evidence of old lesions on lungs, and 5) allergy or, unable to perform EC antigen testing.

As a preliminary exploratory investigation, patients with ATB enrolled in our study had received a diagnosis of lung, tracheal, bronchial, or pleural TB using the Diagnostic Criteria for Tuberculosis (WS288-2017) issued by the National Health and Family Planning Commission. The diagnosis of ATB was established by factoring in epidemiological history, clinical features, chest imaging, and pathogenic examination (that included bacteriology and molecular biology). Relevant exclusion criteria for ATB included the following: 1) history of steroid use; 2) immune function impairment resulting from conditions like post-transplantation, autoimmune disorders, or HIV infection; 3) individuals under 12 years of age; 4) a history of receiving anti-TB medication for more than one month; and 5) individuals who were malnourished.

The inclusion criteria for individuals with LTBI in this study included the following: 1) a documented history of close contact with ATB patients, or employment as staff in a TB-specialized hospital or laboratory; 2) no clinical signs or symptoms suggestive of ATB; 3) normal findings on chest imaging; 4) positive results on IGRAs; 5) confirmed negative HIV status; and 6) aged 12 years or older. Conversely, the exclusion criteria for individuals with LTBI were as follows: 1) any evidence of active TB disease; 2) currently lactating or pregnant women; 3) confirmed positive HIV status; 4) a history of receiving anti-TB medication for more than one month; and 5) individuals below the age of 12.

### 2.3 Candidate antigens determination

The selection of candidate antigens for epitope prediction in this study was based on an extensive evaluation of their performance in both animal models and clinical trials, highlighting their potential as TB vaccine candidates (37). The 19 chosen antigens, namely Ag85A, Ag85B, CFP10, CFP21, ESAT6, EspA, EspC, Mpt51, Mpt63, Mpt64, Mtb8.4, Mtb32a, PPE18, PPE44, PPE68, RpfA, RpfB, RpfE, and TB10.4, have demonstrated encouraging outcomes in previous investigations.

To ensure the reliability of the study’s findings, only antigens meeting strict eligibility criteria were considered for analysis. The original sequences corresponding to these selected antigens were obtained from the NCBI database (https://www.ncbi.nlm.nih.gov/), a reputable source for molecular information. These authentic antigen sequences served as the foundation for subsequent screening and identification of immunodominant epitopes, thereby enabling a comprehensive assessment of their potential in eliciting immune responses.

### 2.4 Screening of eligible T-cell epitopes

To predict HTL and CTL epitopes and their ability to bind to major histocompatibility complex II (MHC II) and MHC I, respectively, we utilized the MHC II server (http://tools.iedb.org/mhcii/) and MHC I server (http://tools.iedb.org/mhci/). Parameter settings for MHC I server: prediction method = IEDB recommendation 2020.09 (NetMHCpan EL4.1), MHC source species = human, MHC allele = HLA allele reference set, epitope length = all lengths. Parameter settings for MHC II server: prediction method = IEDB recommendation 2.22, MHC source species = human, MHC allele = all human leukocyte antigen (HLA) reference sets (HLA-DR, HLA-DP, HLA-DQ), epitope length = 15. HTL and CTL epitopes with a percentile rank < 0.5 were deemed eligible peptides (18). We assessed the antigenicity of HTL and CTL epitopes using the VaxiJen v2.0 server (http://www.ddg-pharmfac.net/vaxijen/VaxiJen/VaxiJen.html), and only epitopes with antigenicity scores > 0.7 were considered for further analysis (38). Next, we predicted the allergenicity and toxicity of these epitopes using the AllerTOP v.2.0 server (http://www.ddg-pharmfac.net/AllerTOP/), Allergen FP v.1.0 server (http://ddg-pharmfac.net/AllergenFP/), and Toxin Pred server (http://crdd.osdd.net/raghava/toxinpred/) tools, respectively. We also utilized the IFN epitope server (http://crdd.osdd.net/raghava/ifnepitope/index.php) and the Class I Immunogenicity server (http://tools.iedb.org/immunogenicity/) to predict the IFN-γ inducibility of HTL epitopes and the immunogenicity of CTL cell epitopes, respectively. We only selected HTL epitopes with positive IFN-γ inducibility and CTL cell epitopes with an immune score of Class I Immunogenicity > 0 (where a higher score can induce a more robust immune response) and a percentile rank of <0.5 for further analysis.

Furthermore, to identify immunodominant HTL and CTL epitopes, the candidate epitopes were evaluated based on multiple criteria. The epitopes with the highest adjusted rank, exceptional antigenicity, and high IFN-γ scores were deemed optimal for further analysis. Also, epitopes with no toxicity or allergenicity were given priority. Identifying epitopes with an outstanding profile across these crucial parameters could elicit a robust and sustained immune response to TB. In addition, to predict the HLA allele-genotypic frequencies in the population, we utilized the Allele Frequency database (http://www.allelefrequencies.net/), which provides comprehensive information on the genetic variation and prevalence of HLA alleles worldwide.

### 2.5 Qualified B-cell epitope prediction

In our study, the accurate prediction of linear B cell epitopes was a crucial step in the design of an effective TB vaccine. Linear B cell epitopes have the potential to induce a humoral immune response and stimulate the production of TB-specific antibodies. To achieve this, we employed the ABC pred server (https://webs.iiitd.edu.in/raghava/abcpred/) for the prediction of linear B cell epitopes. The ABC pred server provided us with epitope scores for each identified epitope, which served as an indicator of the potential strength of the immune response that the epitope could elicit (39). Based on these scores, we selected the most promising epitopes as vaccine candidates. The parameter settings of the ABC pred server were as follows: epitope length was set to 20, and the screening threshold was set at 0.51. It is important to note that the range of thresholds available for selection ranges from +0.1 to +1.0. Increasing the threshold value enhances specificity but may result in decreased sensitivity. Following the screening process described above, the epitopes that successfully passed the threshold criteria were considered as immunodominant B cell epitopes in our analysis, and epitopes with high scores were given priority.

### 2.6 MEV construction

To optimize the antigenicity, immunogenicity, and immunopotency of the MEV, three strategies were employed in this study: selection of linkers, incorporation of TLR (Toll-like receptor) agonists, and inclusion of adjuvant peptides (**Figure S1**M). Firstly, for enhanced stability, expression, and biological activity of the MEV, specific linkers were utilized to connect different components. The rigid linker “EAAAK” was chosen to link TLR agonists and adjuvant peptides, ensuring a stable connection. Similarly, the rigid linker “AAY” was employed to connect CTL epitopes, while the flexible linker “GPGPG” was used for linking HTL epitopes. Additionally, the “KK” linker was used to connect B-cell epitopes. Secondly, to maximize immunogenicity and enhance the targeting of antigen-presenting cells, the MEV was designed to incorporate two TLR agonists. The TLR-2 agonist PorB (IALTLAALPVAAMADVTLYGTIKAGVETSRSVAHNGAQAASVETGTGIVDLGSKIGFKGQEDLGNGLKAIWQVEQ) from *Neisseria meningitidis* was included to stimulate TLR-2 signaling (1-75 aa in MEV), while the TLR-4 agonist RS-09 (APPHALS) from *Lactobacillus plantarum* was incorporated to activate TLR-4 signaling (716-722 aa in MEV). These agonists served to enhance immune responses and improve the delivery of the MEV to immune cells. Thirdly, an auxiliary peptide named Pan HLA DR-binding epitope (PADRE, AGLFQRHGEGTKATVGEPV) was included in the MEV construction. This peptide assists in further augmenting the immunogenicity of the MEV, aiding in the activation of HLA DR-restricted T cells. Finally, to facilitate the purification of the final MEV product, a 6×His tag (HHHHHH) was added to the end of the MEV amino acid sequence. This tag enables the use of nickel affinity chromatography for efficient purification of the MEV during the production process. Furthermore, we also performed BLAST analysis to exclude epitopes recognized by humans using the Protein BLAST (https://blast.ncbi.nlm.nih.gov/Blast.cgi). This step was carried out to avoid potential autoimmune reactions.

### 2.7 Prediction of population coverage, physicochemical, and immunological properties of MEV

The use of vaccines in populations is greatly influenced by the degree of coverage afforded by the HLA-I and HLA-II alleles that restrict the dominant epitopes comprising the vaccine. Hence, in this study, we undertook a comprehensive analysis of the population coverage of HLA-I and HLA-II alleles that restrict the dominant CTL and HTL epitopes of the MEV, respectively, making use of the IEDB database (http://tools.iedb.org/population). To further assess the characteristics of the MEV, our study employed computational methods to evaluate its physicochemical properties, solubility, and antigenicity. Specifically, the expasy server (https://web.expasy.org/protparam/) and the NovoPro server (https://www.novopro.cn/tools/prot-sol.html) were utilized to predict physicochemical properties and solubility, whereas antigenicity was assessed using both the VaxiJen v2.0 server and the ANTIGENpro server. Moreover, to evaluate the safety and potential efficacy of the MEV, further computational analyses were performed to predict its immunogenicity, allergenicity, and toxicity. Immunogenicity was determined using the IEDB Immunogenicity server, while the potential for allergenicity was assessed via the AllerTOP v.2.0 server and the Allergen FP v.1.0 server. In addition, any potential toxicity of the vaccine was predicted by employing the Toxin Pred server.

### 2.8 Prediction of secondary/tertiary structures of the MEV

To predict the secondary structure proportions of the newly designed MEV, the Prabi server was utilized. This server provides the GOR4 method for secondary structure prediction (https://npsa-prabi.ibcp.fr/cgi-bin/npsa_automat.pl?page=/NPSA/npsa_gor4.html). For the prediction of the transmembrane region and signal peptide portion of the vaccine, the NovoPro server was employed. The NovoPro server offers tools for signal peptide prediction (https://www.novopro.cn/tools/signalp) and transmembrane helix prediction (https://www.novopro.cn/tools/tmhmm.html). To predict the tertiary structure of the MEV, the I-TASSER server was utilized. I-TASSER (Iterative Threading ASSEmbly Refinement) is a widely used method for protein structure prediction (https://zhanggroup.org//I-TASSER/). The model with the highest C-score was chosen as the representative structure. To further optimize the predicted tertiary structure, the GalaxyRefine web server was employed (https://galaxy.seoklab.org/cgi-bin/submit.cgi?type=REFINE). This server refines and improves the structural quality of the model derived from I-TASSER predictions. The PyMOL software (Version 2.1.0, SourceForge Headquarters, San Diego, USA) was used for visualizing and editing the Protein Data Bank (PDB) files of the MEV. To assess the accuracy of the predicted tertiary structure, the ProSA-web server was utilized (https://prosa.services.came.sbg.ac.at/prosa.php). The ProSA-web server calculates a Z-score, where a Z-score above zero indicates the potential presence of errors or instability within the protein model. Additionally, the ERRAT server (https://saves.mbi.ucla.edu/) was used to evaluate the quality of the model based on the Ramachandran diagrams, which indicate the allowed regions for amino acid conformations in a protein structure.

### 2.9 Analysis of molecular docking and docking plane interactions between MEV and TLRs

To facilitate the investigation of the TLR2 and TLR4, protein data bank (PDB) structure files were secured from the NCBI Molecular Modeling Database (MMDB) (https://www.ncbi.nlm.nih.gov/structure/). Subsequently, the PDB files were visualized and edited using the PyMOL software (Version 2.1.0, SourceForge Headquarters, San Diego, USA). Molecular docking was then carried out using the ClusPro2.0 server (https://cluspro.bu.edu/home.php). Finally, the 2D ligand-protein interaction diagrams were analyzed using the LigPlot+ software (Version 2.2, European Bioinformatics Institute, Cambridge, United Kingdom) based on JAVA software. These methods provide a comprehensive platform for the holistic study of TLR2 and TLR4 and their interactions with specific ligands.

### 2.10 Recombinant plasmid construction

The amino acid sequence was optimized by the Optimizer server (https://genomes.urv.es/OPTIMIZER/) for codon optimization. Then, the pET28a plasmid was cleaved using NheI and XhoI restriction enzymes. The pET28a expression vector has a T7 promoter and a lac regulator, which requires a high IPTG starting concentration to initiate practical expression (40). However, the pET30a plasmid type is an E. coli protein with a high expression level, the cloning method has multiple cloning sites, and the vector size is 5422 bp, which are several features that make pET30a more suitable as an expression vector. If the pET 28a expression vector is chosen, the impact caused is theoretically very high. Without antibiotics in the prokaryotic Luria-Bertani (LB) medium, it is easy to lose the growth rate of the plasmid with the exogenous gene much faster than the growth rate of the target bacteria, which eventually affects the expression of the target protein (41). Therefore, we chose the pET30a plasmid capable of high expression of E. coli proteins.

### 2.11 Immune simulation of MEV

Then the optimized nucleotide sequence of the MEV was inserted into the plasmid sequence to construct a recombinant plasmid pET28a-MEV. Finally, the simulated immune responses induced by the MEV were analyzed by the C-ImmSim server (https://150.146.2.1/C-IMMSIM/index.php). The parameters were set as follows: default values were selected for random seed, simulation volume, and simulation step; HLAs were selected: A0101, A0201, B0702, B0801, DRB10101, and DRB1501; vaccine injections were without LPS; and adjuvant = 100. Finally, three MEV injections were administered in human hosts (5-day time step for the first injection; 30-day time step for the second injection; and 60-day time step for the third injection).

### 2.12 *In vitro* expression and purification of MEV

The nucleotide sequence of the MEV was introduced into the pET28a (+) plasmid by NheI and XhoI restriction sites, and the recombinant plasmid was transformed into the *Escherichia coli* (*E. coli*) BL21 for expression *in vitro*. Transformed *Escherichia coli* BL21 (DE3) was incubated on LB solid plates (100 µg/mL kanamycin) overnight at 37°C. Individual colonies were picked out, inoculated into liquid LB medium (100 µg/mL kanamycin), and incubated overnight at 37°C and 220 rpm. The first-generation strain (1 mL) was added to liquid LB medium (100 µg/ml kanamycin, 100 ml, 37°C, 220 rpm) and incubated for 4 to 6 h. Then, the first-generation bacteria were inoculated at 1% into a new 1 L volume of LB liquid medium (15 µg/ml kanamycin) and incubated at 37°C and 220 rpm. Next, 0.1 mM IPTG was added to the LB medium and incubated at 16°C and 220 rpm overnight. The bacteria were centrifuged at 8000 rpm for 10 min to collect the cells and resuspended by adding bacterial crushing solution (weight/volume = 1:15). After crushing twice with a high-pressure homogenizer, the cells were centrifuged at 8,000 rpm for 45 min at 4°C, and the supernatant was collected. Finally, the MEV was purified using Ni-affinity chromatography and analyzed by sodium dodecyl sulfate-polyacrylamide gel electrophoresis (SDS-PAGE) according to our previous studies (17, 42, 43).

### 2.13 Enzyme-linked immunospot assay

Five milliliters of blood sample was collected from HCs (*n* = 21), LTBI individuals (*n* = 25), or ATB patients (*n* = 19). PBMCs from blood samples were isolated using a Human Lymphocyte Separation Medium (Solarbio, Beijing, China, Cat: P8610). Then, 2.5×10^5^ PBMCs in 100 μL of AIM-V medium (Life Technology Invitrogen, California, USA, Cat. No. 087-0112DK) were added into a well of the ELISPOT plate, and 50 μL of MEV (100 μg/ml) or 50 μL of AIM-V medium (negative control) was added into the well-containing PBMCs. The ELISPOT plate was incubated for 24 h at 37°C in a CO_2_ incubator. It is important to note that the plates must not be moved during incubation. Finally, the number of interferon-gamma positive (IFN-γ+) T lymphocytes of HCs, LTBI individuals, and ATB patients was detected using a Human IFN-γ ELISOPT kit (Mabtech AB, Nacka Strand, Sweden, Cat. No. 3420-2APW-10) according to the manufacturer’s instructions.

### 2.14 Cytometric bead assay

Five milliliters of blood samples were collected from a total of 22 subjects: 7 HCs, 8 individuals with LTBI, and 7 individuals with ATB. Then, PBMCs were isolated from the blood sample according to the abovementioned method. The concentration of PBMCs was adjusted to 2.5 × 10^6^ cells/mL using the AIM-V medium. One hundred microliters of PBMCs prepared as described above were added to one well of a 96-well cell culture plate (Mabtech AB, Nacka Strand, Sweden) and incubated with 50 μL of MEV (100 μg/mL) or phosphate-buffered saline (PBS) in a CO2 incubator at 37°C for 48 hours. The mixture of cells and medium was aspirated and then centrifuged at 1000 rpm for 10 minutes to collect the supernatant. The supernatant was gently transferred to another new tube. The levels of interleukin-2 (IL-2), IL-4, IL-6, IL-10, IFN-γ, tumor necrosis factor-α (TNF-α), and IL-17A were determined using a BD CBA Human Th1/Th2/Th17 Cytokine Kit (BD Bioscience, San Diego, USA, Cat: 560484) according to our previous study (17). In addition, ten more healthy volunteers were enrolled, a 5 mL blood sample was collected from each participant, and PBMCs were prepared as described above. Then, 100 μL of PBMCs (2.5 × 10^6^ cells/mL) were added to one well of the 96-well cell culture plate and incubated with 50 μL of PBS in a CO_2_ incubator at 37°C for 48 hours. Finally, Th1/Th2/Th17 cytokine levels were determined using the BD CBA Human Th1/Th2/Th17 Cytokine Kit (BD Bioscience, San Diego, USA, Cat: 560484).

### 2.15 Statistical analysis

Data from both ELISPOT and CBA experiments were statistically analyzed using GraphPad Prism 9.5.0 software (San Diego, CA, USA). Briefly, the numbers of spot-forming cells (SFCs) in the three populations of HCs, LTBI, and ATB were compared using an unpaired t-test or Mann-Whitney test based on normality. The levels of Th1/Th2/Th17 cytokines induced by MEV or PBS in HCs, individuals with LTBI, and patients with ATB were analyzed using a one-way ANOVA test or Kruskal-Wallis test based on normality and homogeneity of variance. In addition, the levels of IL-2, IL-4, IL-6, IL-10, IFN-γ, TNF-α, and IL-17A cytokines were analyzed by principal component analysis (PCA) using GraphPad Prism 9.5.0 software. The potential correlation between cytokine groups was determined using the Pearson r method. Simple linear regression was performed using GraphPad Prism 9.5.0 software for highly correlated cytokines. Data are expressed as mean ± standard error (SEM), and *P*<0.05 was considered a statistically significant difference.

## 3 Results

### 3.1 Identification of immunodominant epitopes, construction, physicochemical properties, and immunological properties of the PP13138R vaccine

**Figure 1:**
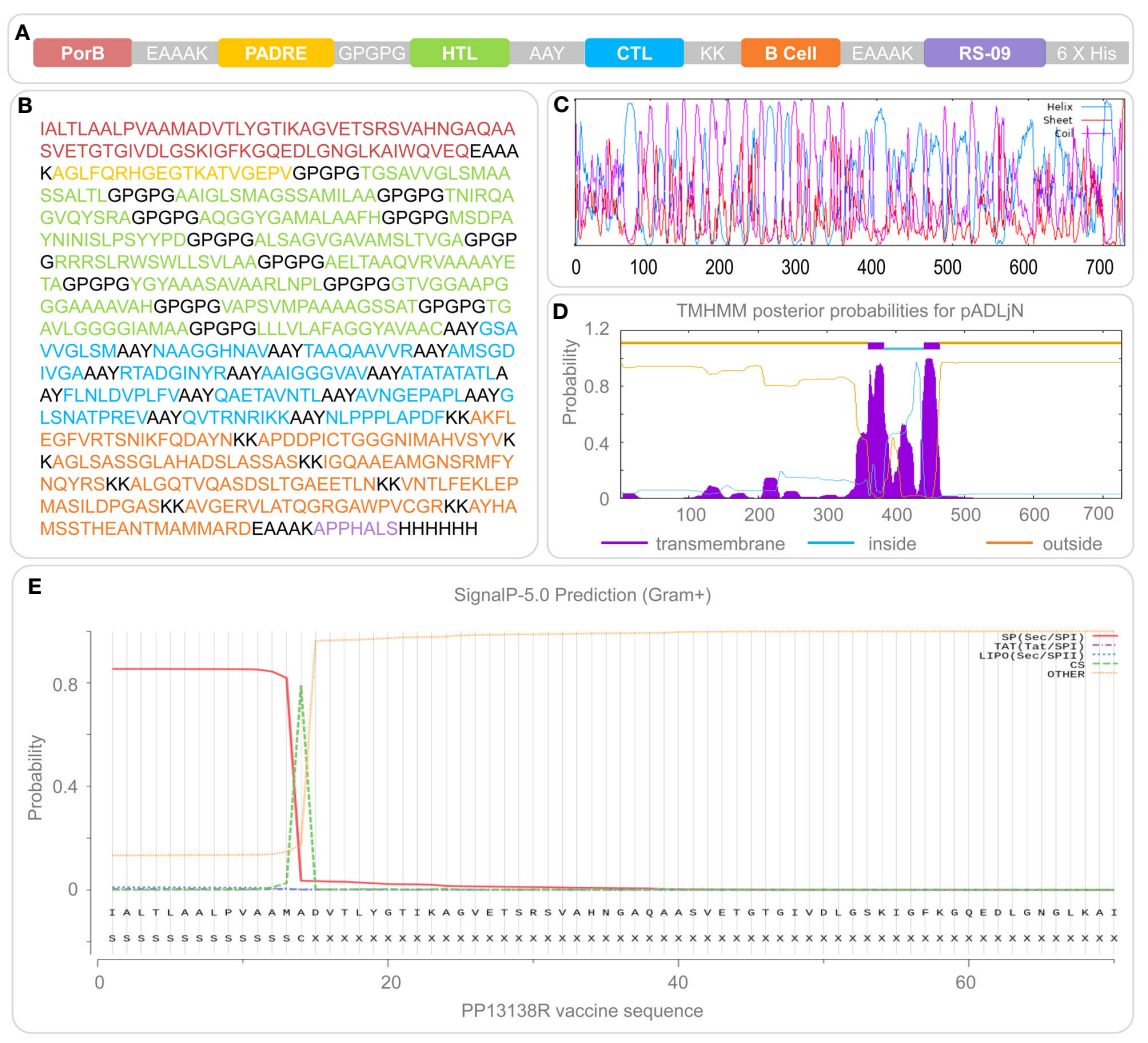
Construction and structural features of the PP13138R vaccine. The PP13138R vaccine was designed with TLR-2 agonist PorB, helper peptide PADRE, HTL epitopes, CTL epitopes, and B-cell epitopes, which are highlighted in different colors in **(A)** and depicted in their corresponding amino acid sequences in **(B)**. The secondary structure of the PP13138R vaccine was predicted using the Prabi server and is shown in **(C)**, where the alpha helix is illustrated in blue, the extended strand in red, and the random coil in purple. The transmembrane region **(D)** and signal peptide **(E)** of the PP13138R vaccine were predicted using the NovoPro server. Inside represents the intracellular region, and higher values indicate a higher likelihood that the amino acid resides within this area. Outside indicates the extracellular region, where next to it is a higher probability that the amino acid exists outside of the cell membrane. The transmembrane signifies the transmembrane section and greater values indicate a higher probability of the amino acid being present in this region.

After epitope prediction and screening, 13 HTL epitopes, 13 CTL epitopes, and 8 B cell epitopes (**Figure 1A**, **Table 1**) were identified as immunodominant epitopes and used to construct a novel MEV, named PP13138R, to fight MTB infection. The sequential arrangement of PP13138R from the N-terminal to C-terminal includes the following elements: PorB agonist, EAAAK linker, PADRE adjuvant peptide, GPGPG linker, HTL immunodominant epitopes, AAY linker, CTL immunodominant epitopes, KK linker, B cell immunodominant epitopes, EAAAK linker, RS-09 agonist, and His purification labels. The PP13138R vaccine comprised 728 amino acid residues (**Figure 1B**), and it’s molecular weight and theoretical PI value were 71565.12 Da and 9.37, respectively. Interestingly, the PP13138R vaccine contains a higher number of positively charged amino acid residues (Arg + Lys) than negatively charged ones (Asp + Glu), which could potentially be important for its function and efficacy in inducing an immune response. One potential reason for this bias toward positive charge is that it could facilitate binding to negatively charged molecules on the surface of antigen-presenting cells (44). This interaction could allow for more efficient uptake and processing of the vaccine by the immune system, leading to a stronger and more targeted immune response. Furthermore, our results showed that the half-life time of the vaccine varies depending on the organism in which it was tested, with the longest half-life time being observed in mammalian reticulocytes (20 hours), followed by *E. coli* (greater than 10 hours) and yeast (30 min). This highlights the importance of understanding the context in which a vaccine was tested, as different organisms may have different physiological processes that affect its stability and effectiveness. Our study also assessed various characteristics of the PP13138R vaccine, including its instability index, aliphatic index, GRAVY score, antigenicity, immunogenicity, and solubility. These indices can provide valuable information about the vaccine’s structure, potential efficacy, and physical properties. Based on our results, the PP13138R vaccine appears to have moderate levels of instability (28.56) and low hydrophobicity, which may be desirable for certain applications. Its high antigenicity (0.8968) and immunogenicity (1.44921) suggest that it could be an effective vaccine candidate, while its moderate solubility score (0.515) may present challenges in formulation and delivery.

**Table 1:** The immunodominant HTL, CTL, and B-cell epitopes were selected to construct the PP13138R vaccine.

Antigen	Allele	Start	End	Length	Peptide	Adjusted rank	IFN-γ scores	Antigenicity Scores	Immunogenicity score	ABC Score
HTL										
Ag85A	HLA-DQA1*06:01,DQB1*03:03	161	178	18	TGSAVVGLSMAASSALTL	0.36	1.1333726	0.8765	NA	NA
Ag85B	HLANADQA1*03:01,DQB1*06:01	161	177	17	AAIGLSMAGSSAMILAA	0.49	1.1091804	0.7215	NA	NA
CFP10	HLANADQA1*03:01,DQB1*06:01	74	86	13	TNIRQAGVQYSRA	0.31	0.61651125	0.7348	NA	NA
MPT51	HLANADQA1*03:01,DQB1*06:01	145	158	14	AQGGYGAMALAAFH	0.33	0.024282193	0.8205	NA	NA
Mpt64	HLANADRB3*02:02	45	62	18	MSDPAYNINISLPSYYPD	0.38	1.024208	1.0403	NA	NA
MTB8.4	HLANADQA1*05:01,DQB1*03:01	7	23	17	ALSAGVGAVAMSLTVGA	0.43	0.59925408	0.9526	NA	NA
MTB32A	HLANADPA1*01,DPB1*04:01	5	20	16	RRRSLRWSWLLSVLAA	0.43	0.59880927	1.2614	NA	NA
PPE18	HLANADQA1*01:02,DQB1*06:02	84	101	18	AELTAAQVRVAAAAYETA	0.18	1.0131456	0.7988	NA	NA
PPE44	HLANADQA1*03:01,DQB1*06:01	152	167	16	YGYAAASAVAARLNPL	0.02	0.69654642	0.7955	NA	NA
PPE44	HLANADQA1*05:01,DQB1*03:01	270	287	18	GTVGGAAPGGGAAAAVAH	0.03	1.148739	1.5999	NA	NA
PPE68	HLANADQA1*03:01,DQB1*06:01	304	319	16	VAPSVMPAAAAGSSAT	0.31	0.23735279	1.0173	NA	NA
RpfA	HLANADQA1*05:01,DQB1*03:01	21	34	14	TGAVLGGGGIAMAA	0.03	0.51780631	0.7682	NA	NA
RpfB	HLANADQA1*05:01,DQB1*03:01	9	24	16	LLLVLAFAGGYAVAAC	0.12	0.26811457	0.7437	NA	NA
CTL										
Ag85A	HLANAC*03:04	162	170	9	GSAVVGLSM	NA	NA	1.0016	0.02032	NA
Ag85B	HLANAB*15:11	285	294	10	NAAGGHNAV	NA	NA	1.9957	0.12765	NA
CFP10	HLANAA*33:03	49	57	9	TAAQAAVVR	NA	NA	0.7677	0.03003	NA
MPT51	HLANAA*02:01	289	297	9	AMSGDIVGA	NA	NA	1.0252	0.18192	NA
Mpt63	HLANAA*11:01	99	107	9	RTADGINYR	NA	NA	1.8872	0.18568	NA
MTB32A	HLANAC*03:04	141	149	9	AAIGGGVAV	NA	NA	1.3251	0.1999	NA
PPE18	HLANAC*01:02	158	166	9	ATATATATL	NA	NA	1.0012	0.1821	NA
PPE44	HLANAA*02:01	232	241	10	FLNLDVPLFV	NA	NA	1.3663	0.09714	NA
PPE68	HLANAC*03:04	155	163	9	QAETAVNTL	NA	NA	0.875	0.16574	NA
RpfA	HLANAC*03:04	141	149	9	AVNGEPAPL	NA	NA	1.1393	0.1456	NA
RpfA	HLANAA*02:01	117	126	10	GLSNATPREV	NA	NA	1.0472	0.10743	NA
RpfB	HLANAA*30:01	191	199	9	QVTRNRIKK	NA	NA	0.7561	0.09342	NA
RpfE	HLANAC*01:02	73	82	10	NLPPPLAPDF	NA	NA	1.441	0.00407	NA
B cell										
Ag85A	NA	269	288	20	AKFLEGFVRTSNIKFQDAYN	NA	NA	NA	NA	0.91
CFP21	NA	178	197	20	APDDPICTGGGNIMAHVSYV	NA	NA	NA	NA	0.91
EspA	NA	256	275	20	AGLSASSGLAHADSLASSAS	NA	NA	NA	NA	0.9
MPT51	NA	243	262	20	IGQAAEAMGNSRMFYNQYRS	NA	NA	NA	NA	0.93
MTB32A	NA	175	194	20	ALGQTVQASDSLTGAEETLN	NA	NA	NA	NA	0.89
PPE68	NA	160	179	20	VNTLFEKLEPMASILDPGAS	NA	NA	NA	NA	0.91
RpfA	NA	97	116	20	AVGERVLATQGRGAWPVCGR	NA	NA	NA	NA	0.92
TB10.4	NA	68	87	20	AYHAMSSTHEANTMAMMARD	NA	NA	NA	NA	0.86

NA, not applicable.

### 3.2 Analysis of the population coverage of the PP13138R vaccine and exclusion of epitopes recognized by humans

Population coverage analysis is crucial in assessing the potential effectiveness of a vaccine candidate in a specific population. Population coverage of the immunodominant CD4^+^ T cell and CD8^+^ T cell epitopes elicited by the PP13138R vaccine was analyzed using data from the IEDB database. Our analysis revealed that: the average coverage of the immunodominant CD8^+^ T cell epitopes in the PP13138R vaccine for HLA-I alleles is 52.42%, and the average coverage of the immunodominant CD4^+^ T cell epitopes in the PP13138R vaccine for HLA-II alleles is 79.69% (**Table 2**). The percentages in the population coverage analysis represent the proportion of individuals within the specified population that are predicted to possess HLA alleles capable of binding to and presenting the selected T cell epitopes. These percentages indicate the potential coverage of the vaccine candidate within that particular population. In addition, BLAST results showed that the Alignment Scores of the 13 HTL, 13 CTL and 8 B-cell epitopes comprising the PP13138R vaccine with human antigens were less than 40, suggesting that immunization of the human body with the PP13138R vaccine does not cause autoimmune diseases.

**Table 2:** Global population coverage of the PP13138R vaccine for HLA I and II alleles.

Population/area	Class I			Class II		
	Coverage ^a^	Average_hit ^b^	Pc90 ^c^	Coverage ^a^	Average_hit ^b^	Pc90 ^c^
Central Africa	31.89%	0.82	0.15	75.00%	1.62	0.4
Central America	1.40%	0.01	0.1	98.31%	5.36	3.48
East Africa	40.83%	1.15	0.17	83.43%	2.16	0.6
East Asia	73.63%	2.38	0.38	67.03%	2.55	0.3
Europe	65.15%	2.14	0.29	69.48%	2.29	0.33
North Africa	46.50%	1.12	0.19	73.52%	2.37	0.38
North America	69.39%	2.32	0.33	86.44%	3.7	0.74
Northeast Asia	81.17%	2.39	0.53	90.13%	3.72	1.01
Oceania	66.73%	1.82	0.3	93.51%	3.83	1.2
South America	45.62%	1.51	0.18	90.26%	4.42	1.05
South Asia	52.68%	0.91	0.21	66.30%	2.23	0.3
Southeast Asia	79.95%	2.56	0.5	81.79%	2.62	0.55
Southwest Asia	47.28%	1.11	0.19	68.41%	2.47	0.32
West Africa	49.18%	1.37	0.2	85.86%	2.32	0.71
West Indies	41.98%	1.09	0.17	72.44%	2.18	0.36
World	67.14%	2.15	0.3	73.08%	2.64	0.37
Average	52.42	1.49	0.25	79.69	2.9	0.76

a projected population coverage.

b average number of epitope hits/HLA combinations recognized by the population.

c minimum number of epitope hits/HLA combinations recognized by 90% of the population.

### 3.3 Analysis of the secondary and tertiary structures of the PP13138R vaccine

Prabi server analysis indicated that the PP13138R vaccine’s secondary structure was composed of 334 α-helices, 75 extended strands, and 319 random coils, which accounted for 45.88%, 10.3%, and 43.82% of the total amino acid count, respectively (**Figure 1C**). Transmembrane prediction results suggested that PP13138R may be a double transmembrane protein with its outer regions located between residues 1 to 359 and 464 to 728 of the amino acid sequence, TMhelix regions located between residues 360 to 382 and 441 to 463 of the amino acid sequence, and an inner region located between residues 383 to 440 of the amino acid sequence (**Figure 1D**). We also investigated potential signal peptides in the PP13138R vaccine and found a 53.461% signal peptide probability with an SP(Sec/SPI) type of signal peptide (**Figure 1E**). The most likely cleavage site was predicted to be located between residues 14-15 of the amino acid sequence of the PP13138R vaccine, with a 25.240% probability of the possible signal peptide sequence being IALTLAALPVAAMA (**Figure 1E**).

**Figure 2:**
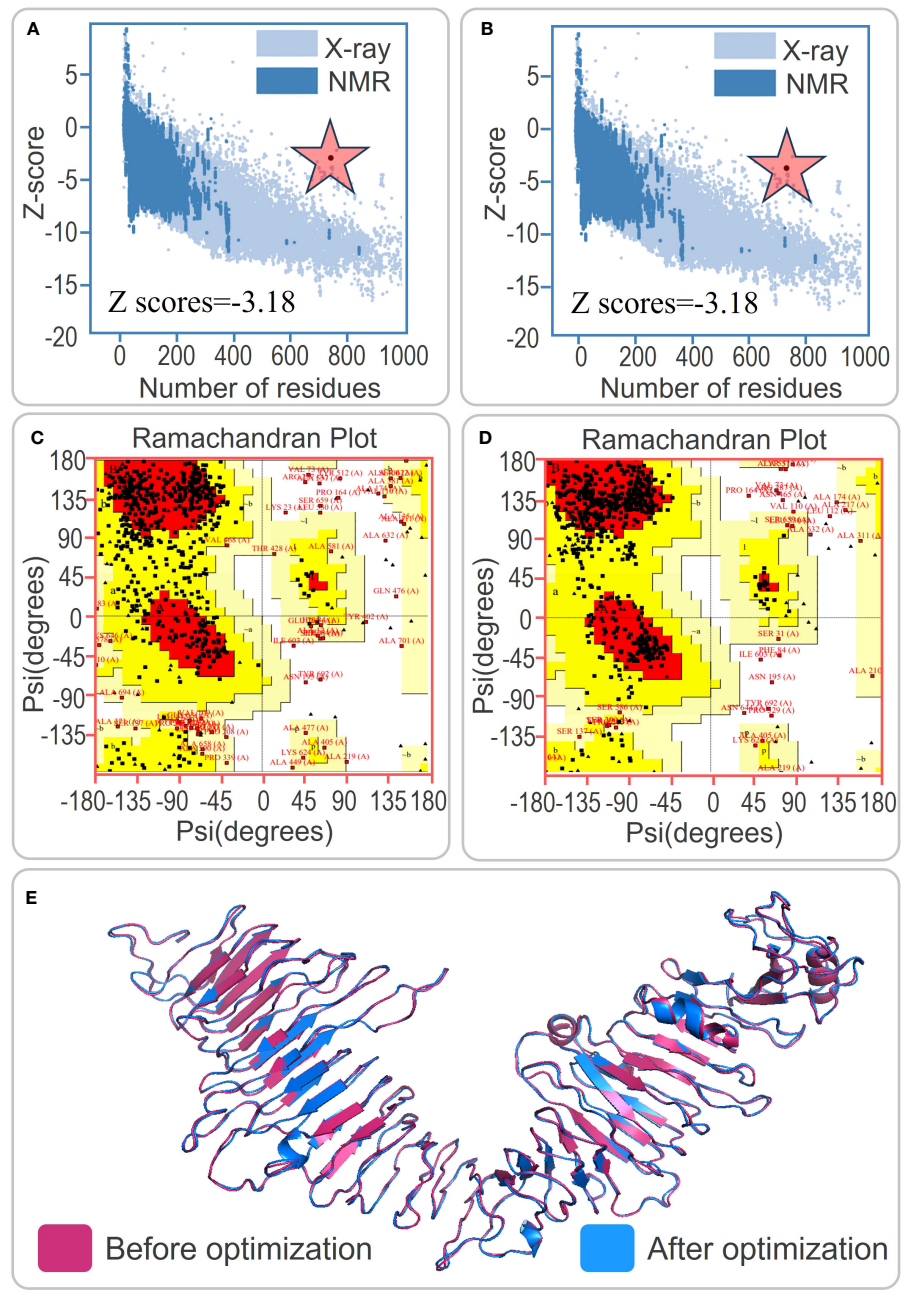
Tertiary structure prediction and optimization of the PP13138R vaccine. **(A)** and **(B)** Z score analysis of the tertiary structure prediction of the PP13138R vaccine before and after optimization. **(C)** and **(D)** Ramachandran plot analysis showing the ratio of core, allow, gener, and disall in the tertiary structure of the PP13138R vaccine before and after optimization. **(E)** Schematic representation of the tertiary structure predicted by the I-TASSER server in pink, and optimized by the Galaxy Refine server in blue. The tertiary structure of the PP13138R vaccine is shown before and after optimization to improve stability and binding ability. The color scheme of the structure indicates the pre-optimized model in pink and the post-optimized model in blue. The Z score and Ramachandran plot analyses demonstrate that the optimized PP13138R vaccine has improved quality compared to the pre-optimized structure. The red star is the z-score.

The present study focused on predicting the tertiary structure of the PP13138R vaccine using bioinformatic tools. The I-TASSER server was utilized to generate five models, with model 1 exhibiting the highest predictive quality, possessing a C-score of -0.88, an estimated TM-score of 0.60 ± 0.14, and an estimated RMSD of 10.2 ± 4.6Å. Five additional models were generated using the Galaxy server for subsequent structural optimization, with higher GDT-HA values and lower MolProbity scores indicating superior model quality. Following optimization, the model exhibiting the highest GDT-HA value and the lowest MolProbity score was selected for further analysis. Notably, the Z scores of the PP13138R vaccine before and after ProSA optimization remained consistent at -3.18 (**Figures 2A, B**). Moreover, the Ramachandran plot generated by the UCLA-DOE LAB SAVES v6.0 server demonstrated that the optimized tertiary structure of the PP13138R vaccine consisted of 72.6% (414) core, 22.1% (126) allow, 2.5% (14) gener, and 2.8% (16) disall (**Figure 2C**), indicating a significant structural improvement over the pre-optimized structure, which contained 56.1% (320) core, 34.7% (198) allow, 6.3% (36) gener, and 2.8% (16) disall (**Figure 2D**). The tertiary structures of the PP13138R vaccine before and after optimization are depicted in **Figure 2E**.

### 3.4 Molecular docking of the PP13138R vaccine with TLRs and interaction analysis of docking planes

**Figure 3:**
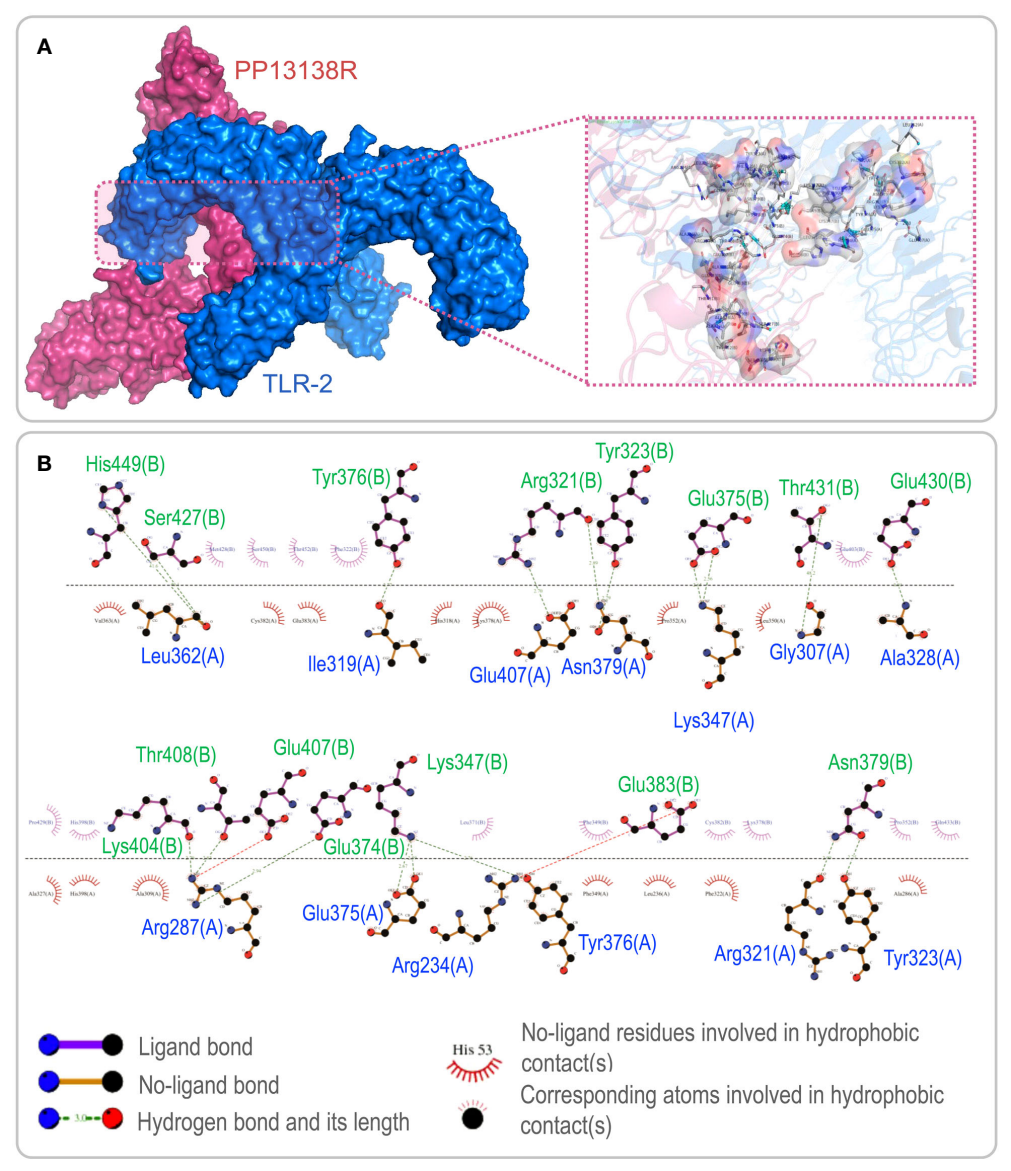
Visualization of molecular docking between the PP13138R vaccine and TLR2. **(A)** Results of molecular docking analysis for PP13138R vaccine with TLR2 predicted by Cluspro Server are shown. The left panel depicts a cartoon diagram of the molecular docking results, while the right panel displays a 3D zoomed-in diagram of the interactions between bonds at the molecular docking site. **(B)** 2D ligand-protein interaction diagram of the PP13138R-TLR2 complex created using the LigPlot^+^ software.

**Figure 4:**
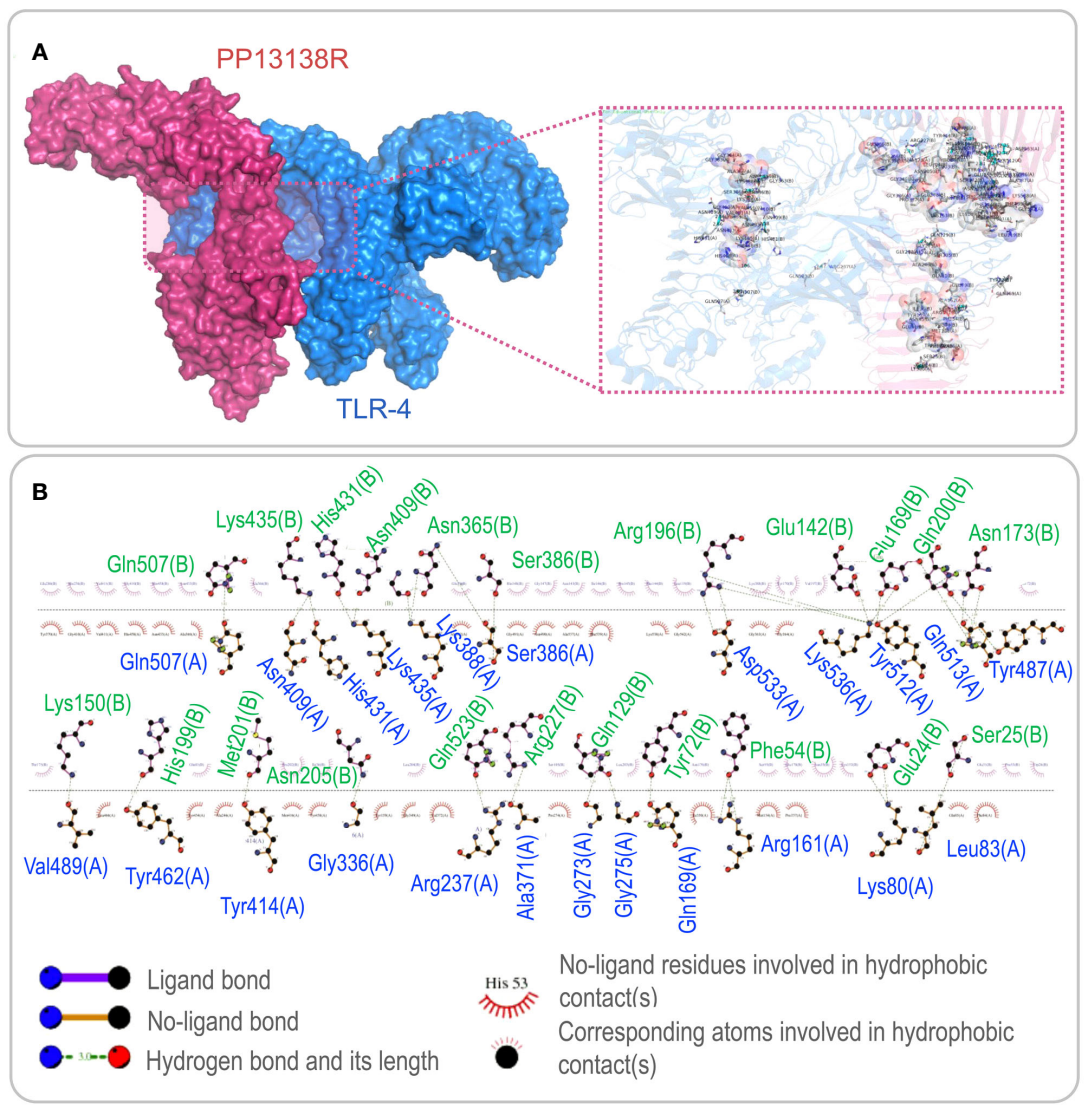
Visualization of molecular docking between the PP13138R vaccine and TLR4. **(A)** Results of molecular docking analysis for PP13138R vaccine with TLR4 predicted by Cluspro Server are shown. The left panel depicts a cartoon diagram of the molecular docking results, while the right panel displays a 3D zoomed-in diagram of the interactions between bonds at the molecular docking site. **(B)** 2D ligand-protein interaction diagram of the PP13138R-TLR4 complex created using the LigPlot^+^ software.

To investigate the interaction between the PP13138R vaccine and TLR2 or TLR4, we utilized the ClusPro2.0 server to generate 30 model complexes. The model complex exhibiting the lowest binding energy was selected for further analysis. Our results demonstrated that the PP13138R vaccine forms a stable complex with both TLR2 and TLR4. Specifically, for the PP13138R-TLR2 complex, the model with a central energy-weighted score of -999.3 kcal/mol and the lowest energy-weighted score of -1167.3 kcal/mol displayed optimal binding affinity (**Figure 3A**). The 2D ligand-protein interaction diagram generated by LigPlot^+^ revealed the presence of 18 hydrogen bonds in the molecular docking complex of PP13138R-TLR2 (**Figure 3B**). In the case of the PP13138R-TLR4 complex, the model exhibiting a central energy-weighted score of -1111.5 kcal/mol and the lowest energy-weighted score of -1255.1 kcal/mol exhibited the most favorable binding affinity (**Figure 4A**). The 2D ligand-protein interaction diagram obtained from LigPlot^+^ demonstrated 29 hydrogen bonds in the molecular docking complex of PP13138R-TLR4 (**Figure 4B**). These findings support the notion that the PP13138R vaccine interacts with both TLR2 and TLR4 in a stable and specific manner.

### 3.5 Immune responses induced by the PP13138R vaccine *in silico*

**Figure 5:**
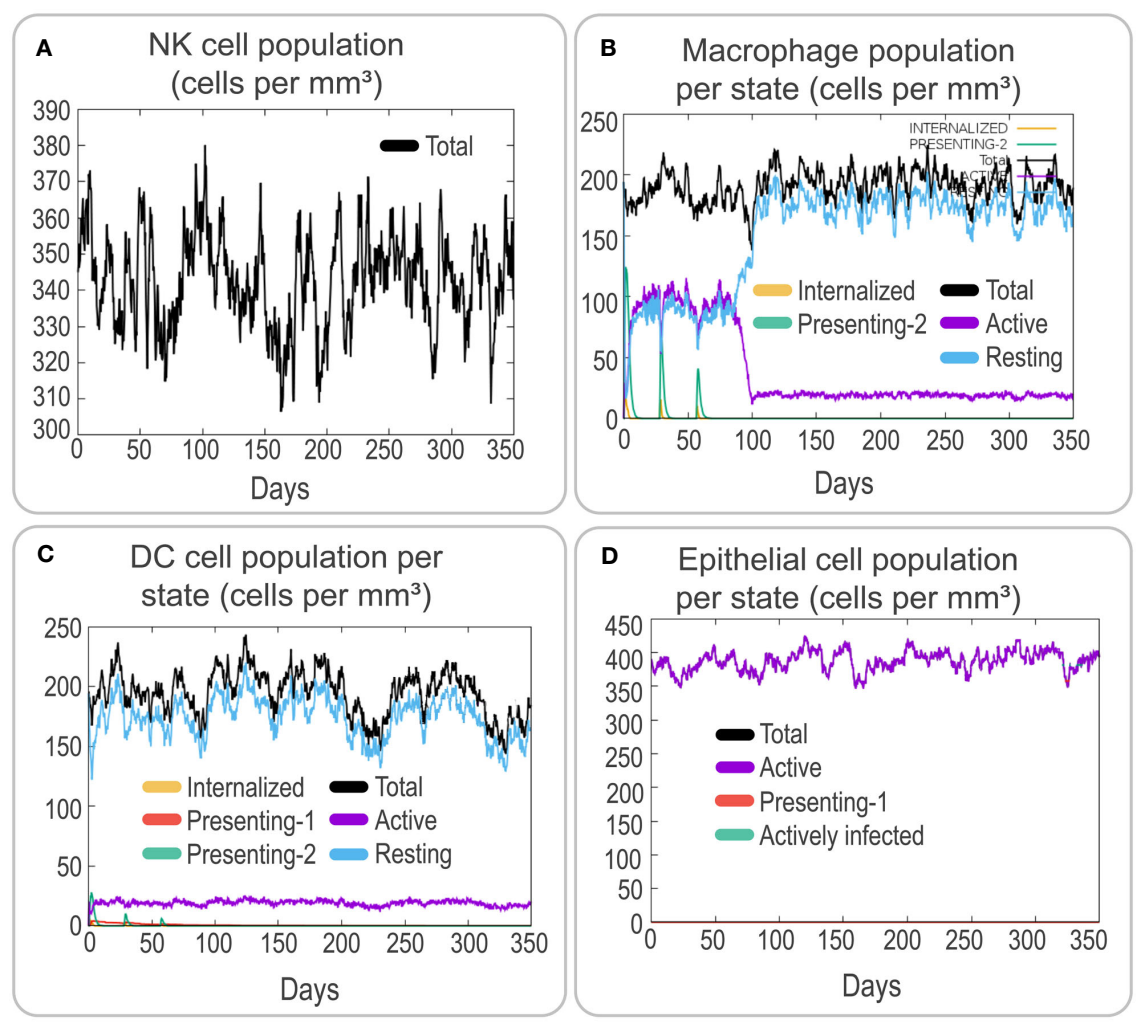
Analysis of innate and endothelial immune responses induced by the PP13138R vaccine using C-ImmSim. The potential immunological effects of the PP13138R vaccine were evaluated using C-ImmSim. Simulation of three immunizations with the PP13138R vaccine in the C-ImmSim server allowed observation of the number of NK cells **(A)**, macrophages per state **(B)**, DCs per state **(C)**, and epithelial cells per state **(D)**. NK cells stand for nature killer cells, and DCs represent dendritic cells.

**Figure 6:**
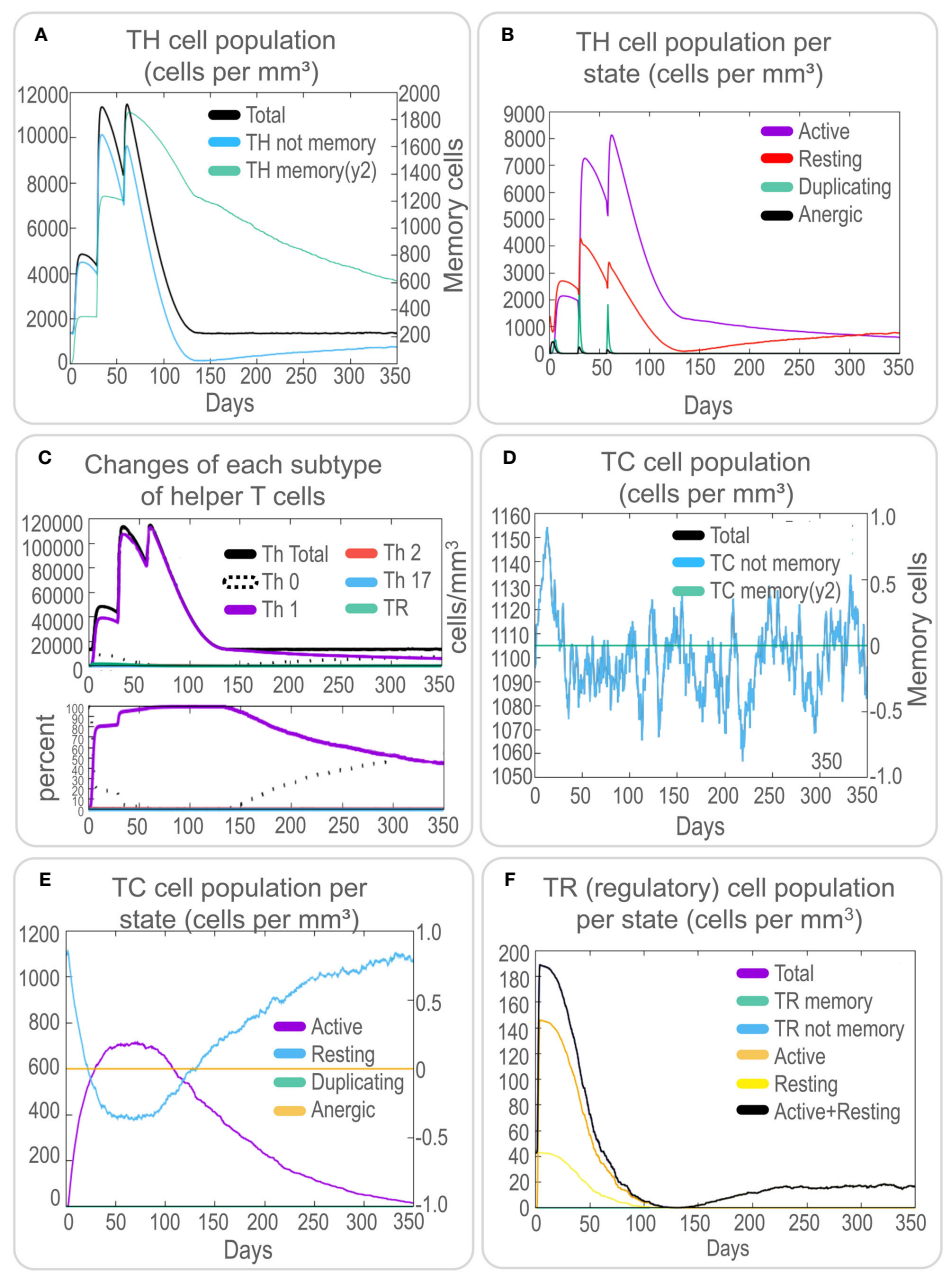
*In silico* analysis of T cell immune responses induced by the PP13138R vaccine using C-ImmSim. To evaluate the potential T cell immune responses induced by the PP13138R vaccine, an *in silico* analysis was performed using C-ImmSim. The results indicate the number of TH cells **(A)**, TH cell population per state **(B)**, changes in each subtype of helper T cells **(C)**, TC cell population **(D)**, TC cell population per state **(E)**, and TR (regulatory) cell population per state **(F)**. TH refers to helper T lymphocytes, TC represents cytotoxic T lymphocytes, and TR denotes regulatory T lymphocytes.

To assess the immune response elicited by the PP13138R vaccine, we utilized the C-ImmSim server. Our findings demonstrate that the vaccine can stimulate a strong immune response, as evidenced by the elevated levels of various immune cells. Notably, the PP13138R vaccine induced higher levels of NK cells (approximately 340 cells per mm³, **Figure 5A**), active macrophages (90 cells per mm³ at 10-80 days, **Figure 5B**), active dendritic cells (DCs) (around 20 cells per mm³, **Figure 5C**), and active epithelial cells (approximately 400 cells per mm³, **Figure 5D**). Further analysis revealed that three immunizations with the PP13138R vaccine induced three sequential peaks of presenting-2 macrophages (**Figure 5B**) and presenting-2 DCs (**Figure 5C**). Moreover, the PP13138R vaccine was found to elicit robust adaptive immune responses, with three peaks of memory (2×10^3^ cells/mm^3^, 7.5×10^3^ cells/mm^3^, and 1.1×10^4^ cells/mm^3^, **Figure 6A**) and non-memory helper T (Th) cells (4.5×10^3^ cells/mm^3^, 1×10^4^ cells/mm^3^, and 9.5×10^3^ cells/mm^3^, **Figure 6A**), as well as active Th cells (2×10^3^ cells/mm^3^, 7.2×10^3^ cells/mm^3^, and 8.1×10^3^ cells/mm^3^, **Figure 6B**), resting Th cells (2.8×10^3^ cells/mm^3^, 4.2×10^3^ cells/mm^3^, and 3.3×10^3^ cells/mm^3^, **Figure 6B**), duplicating Th cells (500 cells/mm^3^, 2.5×10^3^ cells/mm^3^, and 2×10^3^ cells/mm^3^, **Figure 6B**), and Th1 type T lymphocytes (4×10^4^ cells/mm^3^, 1.1×10^5^ cells/mm^3^, and 1.2×10^5^ cells/mm^3^, **Figure 6C**) induced by three doses of the vaccine. The induction of adaptive immunity *in silico* was good overall and the results showed a predominance of Th2-type immune responses.

Furthermore, the PP13138R vaccine effectively stimulated an increase in non-memory TC cell numbers to 1150 cells/mm^3^ (**Figure 6D**) and active TC cell numbers to 750 cells/mm^3^ (**Figure 6E**) after the first immunization. In addition, the vaccine-induced regulatory T-cell counts rapidly peaked at 190 cells/mm^3^ after the first immunization (**Figure 6F**). These results suggest that the PP13138R vaccine can induce a strong and coordinated immune response, which may be critical for its efficacy as a potential vaccine candidate.

**Figure 7:**
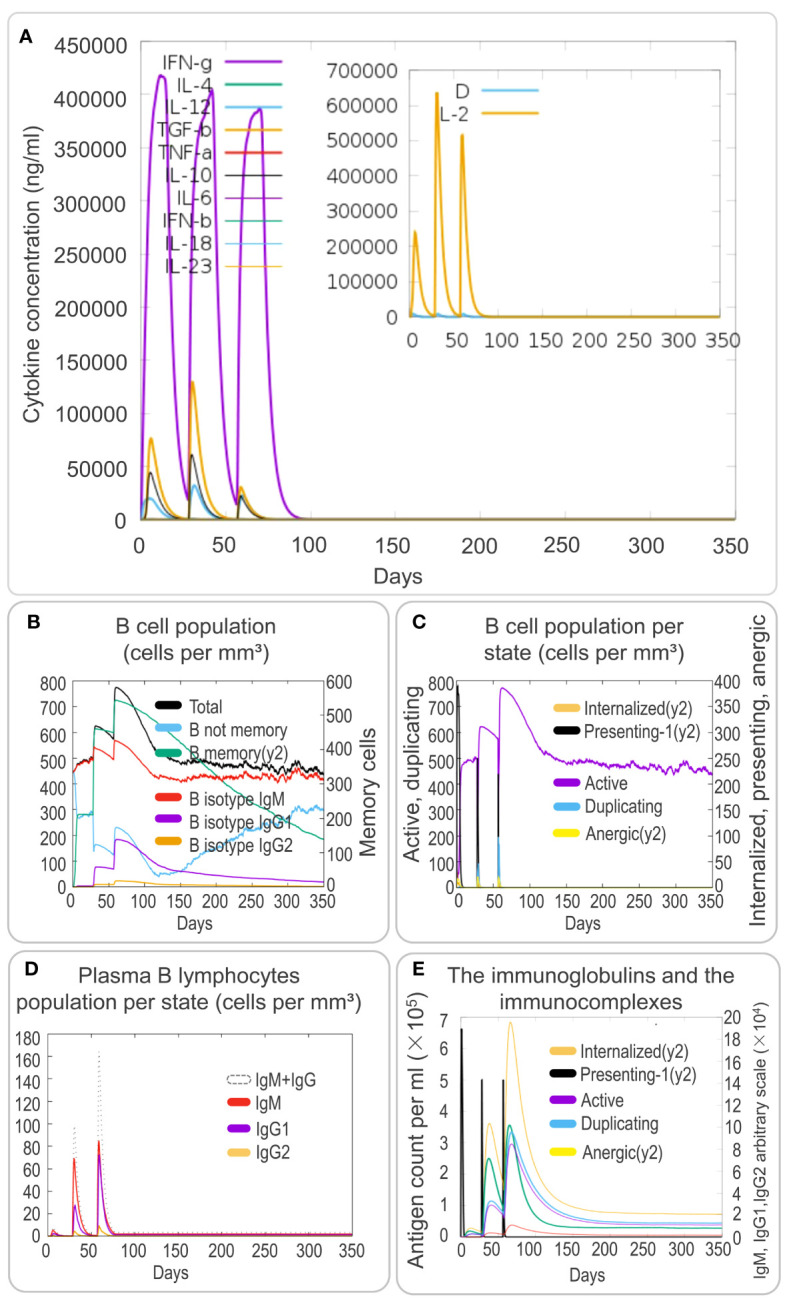
*In silico* analysis of cytokines and B-cell immune responses induced by the PP13138R vaccine using C-ImmSim. To analyze the cytokine responses and B-cell immune response induced by the PP13138R vaccine, an *in silico* analysis was conducted using C-ImmSim. Dynamic changes in cytokines such as IFN-γ, IL-4, TGF-β, TNF-α, IL-10, IL-6, IFN-β, IL-18, and IL-23 were assessed after three immunizations with the PP13138R vaccine **(A)**. Additionally, the PP13138R vaccine was used to evaluate its potential to induce different types of B cell populations **(B)**, B-cell populations per state **(C)**, plasma B-lymphocyte populations per state **(D)**, and immunoglobulins and immune complexes **(E)** after three simulated immunizations in C-ImmSim.

To investigate the cytokine profile induced by the PP13138R vaccine, we analyzed the *in silico* data. We found that the vaccine could generate high levels of IFN-γ, TGF-β, IL-10, and IL-12 production by immune cells. These cytokines formed three peaks, suggesting a coordinated immune response elicited by the vaccine (**Figure 7A**). The three peaks of IFN-γ after three stimuli were 4.2×10^5^, 4.0×10^5^, 3.8×10^5^; the three peaks of TGF-β were 7.5×10^4^, 1.25×10^5^, 3.0×10^4^; the three peaks of IL-2 were 2.5×10^5^, 6.3×10^5^, 5.1×10^5^; and the peaks of other cytokines ranged between 0 and 2.5×10^4^; D2 aggregates were maintained at very low levels (**Figure 7A**). Moreover, the PP13138R vaccine elicited a strong humoral immune response. Specifically, it induced a significant increase in the number of memory B lymphocytes, B isotype IgM, and B isotype IgG1 (**Figure 7B**) and active, presenting-1, and duplicating B cells (**Figure 7C**), with three peaks observed. Furthermore, the vaccine stimulated plasma B lymphocytes to produce significantly high levels of IgM and IgG1 antibodies (**Figure 7D**) and induced high levels of immunoglobulins and immunocomplexes (**Figure 7E**). These data suggest that the PP13138R vaccine is capable of inducing both Th1 and Th2 type immune responses, especially Th2 type immune responses. Overall, these findings indicate that the PP13138R vaccine can generate both cellular and humoral immune responses, which may be essential for its potential effectiveness as a vaccine candidate (**Supplementary Materials S1**).

### 3.6 *In vitro* cloning and expression of the PP13138R vaccine and real-world evaluation of its immunological properties

**Figure 8:**
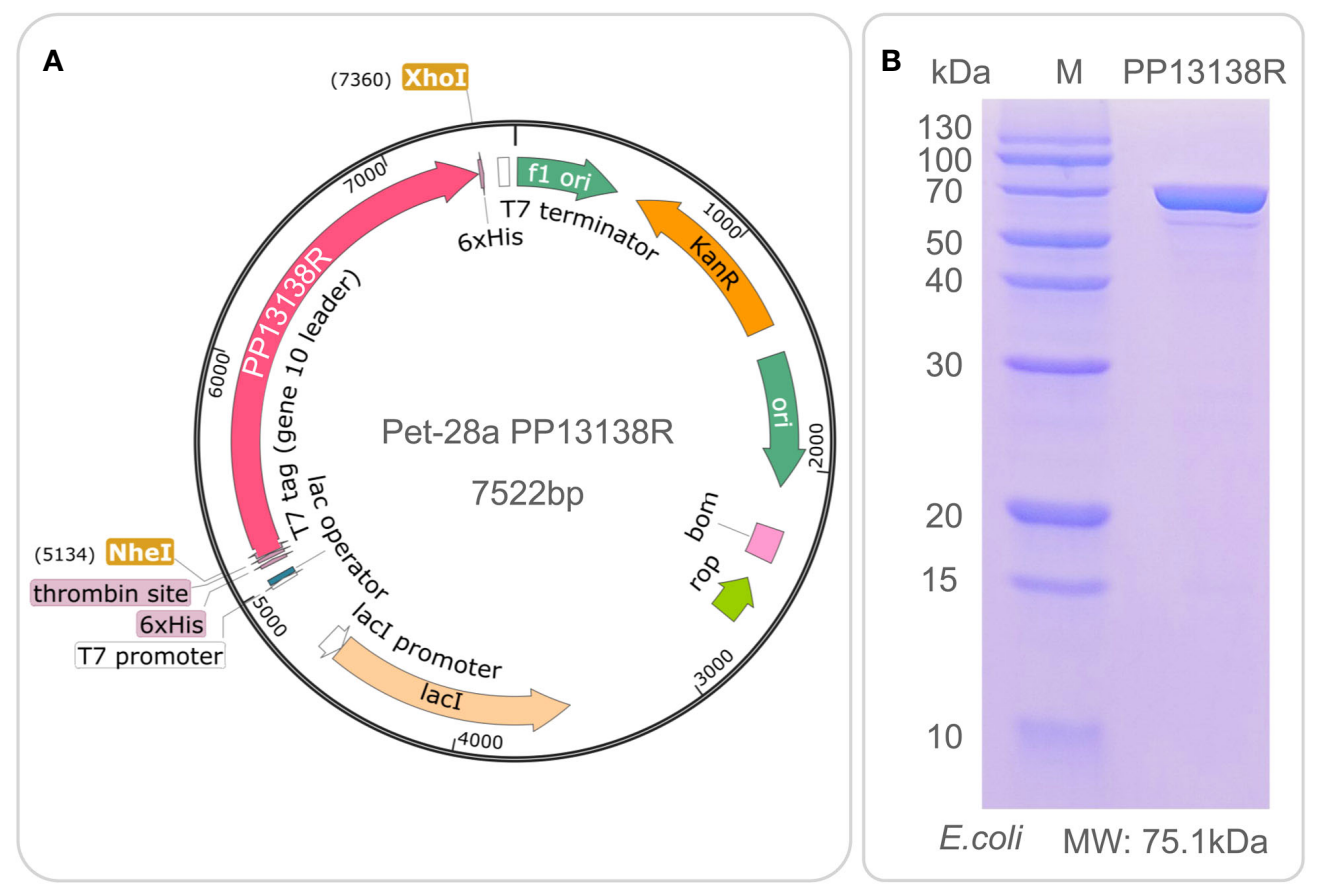
Construction of pET28a-PP13138R recombinant plasmid and expression and purification of the PP13138R vaccine *in vitro*. The codon-optimized gene sequence of the PP13138R vaccine was inserted into the genome of the pET28a plasmid via NheI and XhoI double digestion sites to construct the pET28a-PP13138R recombinant plasmid **(A)**. The recombinant plasmid was introduced into the *E*. *coli* expression vector to express the PP13138R vaccine, purified by 6× His-tag adsorption on a nickel column, and finally identified by SDS-PAGE **(B)**.

**Figure 9:**
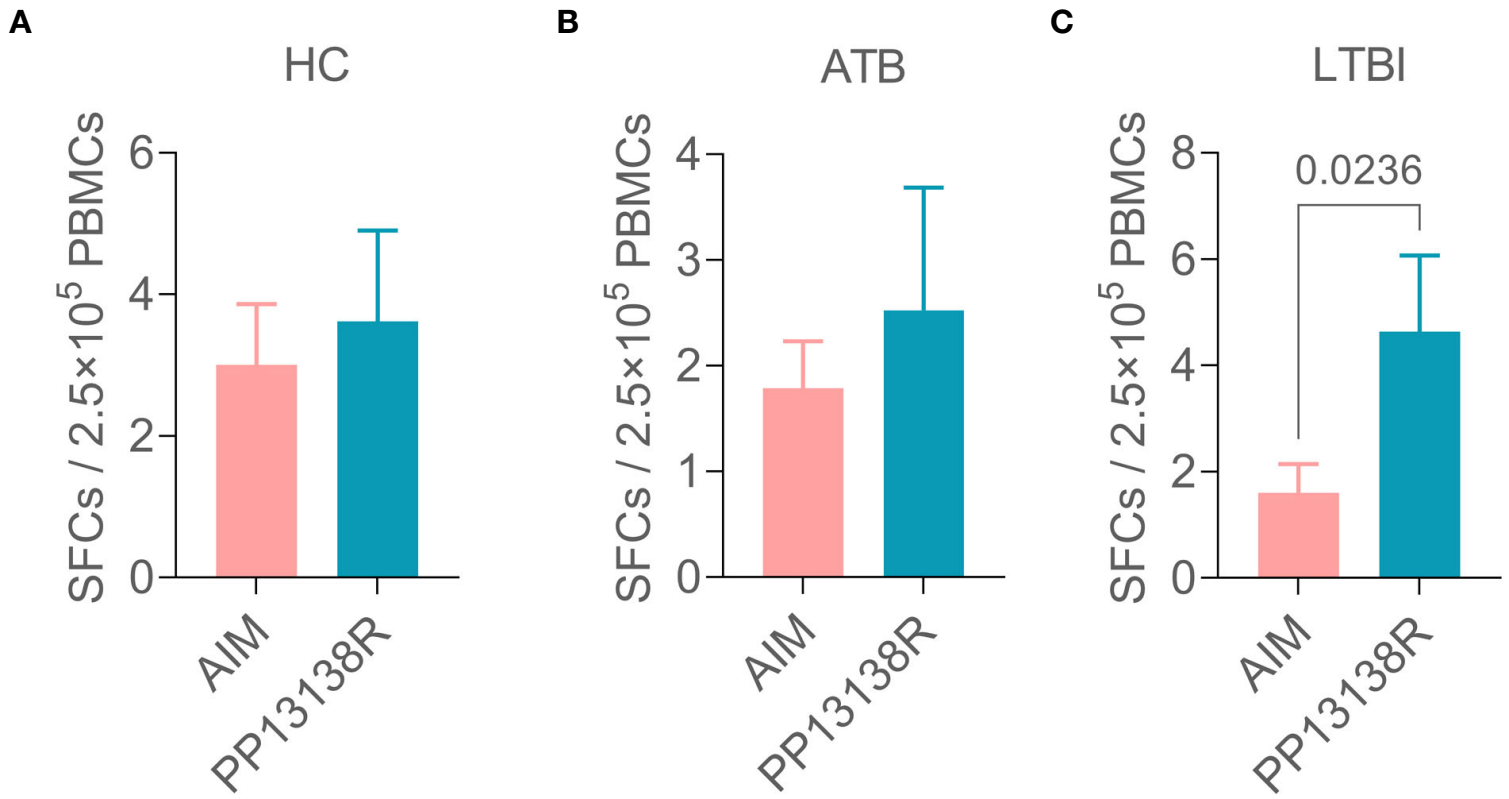
Differences in the number of IFN^+^ T lymphocytes induced by the PP13138R vaccine in HCs, ATB patients, and LTBI individuals. PMBCs collected from HCs **(A)**, ATB patients **(B)**, and LTBI individuals **(C)** were stimulated with the PP13138R vaccine or AIM-V medium, and the number of IFN^+^ T lymphocytes were detected using ELISPOT. The data were shown as mean + SEM and analyzed using the Unpaired *t*-test or Mann Whitney test according to the normality. *P*<0.05 was considered significantly different. SEM, standard error of the mean; SFCs, spot-forming cells.

**Figure 10:**
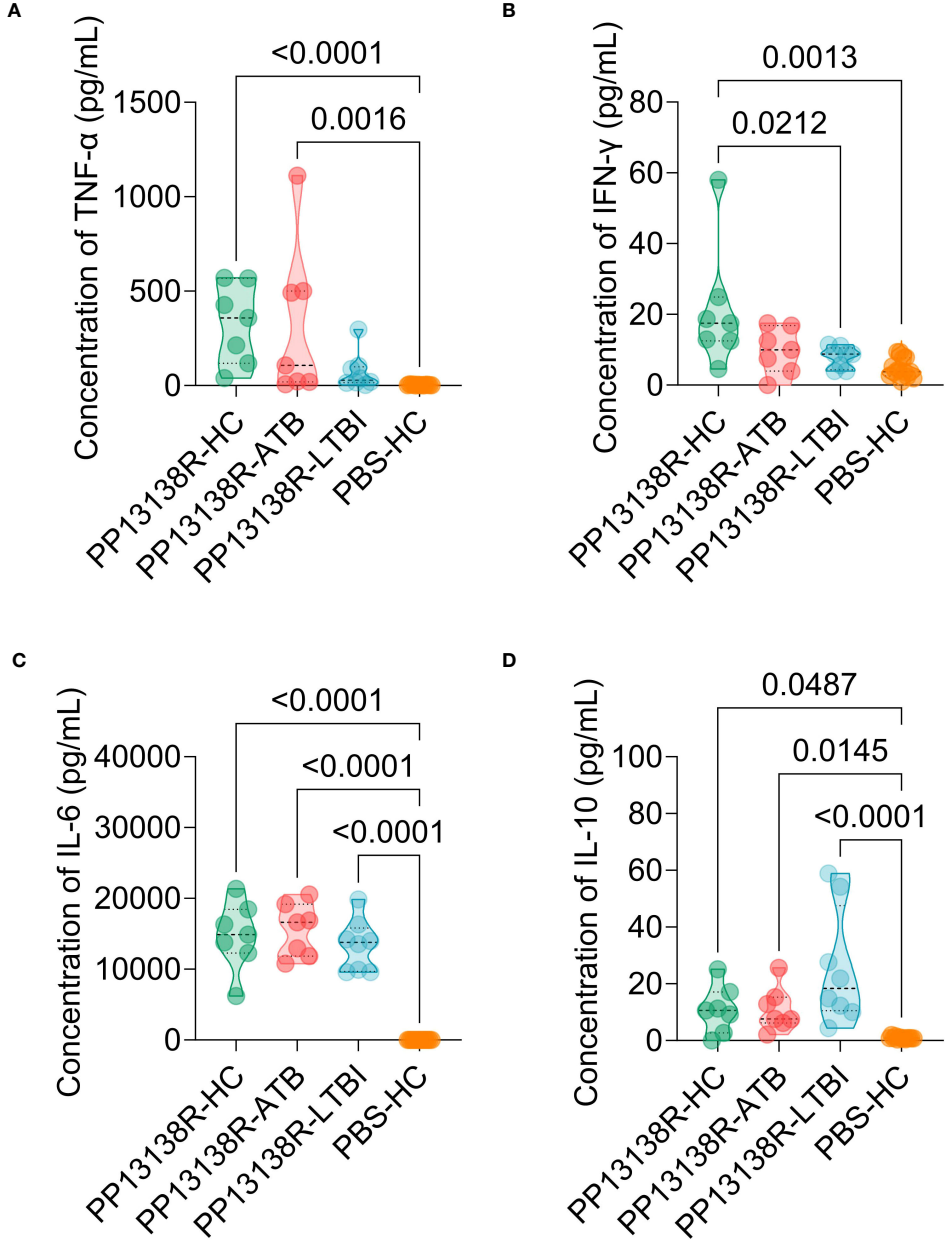
Differences in the levels of cytokines induced by the PP13138R vaccine in HCs, ATB patients, and LTBI individuals. PMBCs collected from HCs, ATB patients, and LTBI individuals were stimulated with the PP13138R vaccine or AIM-V medium, and the levels of TNF-α **(A)**, IFN-γ **(B)**, IL-6 **(C)**, and IL-10 **(D)** were detected using a Human Th1/Th2/Th17 Cytokine Kit. The results were analyzed using the one-way analysis of variance (ANOVA) or Kruskal-Wallis test according to the data normality and homogeneity of variances. All data were shown as mean + SEM. *P*<0.05 was considered significantly different. SEM, standard error of the mean.

It is interesting to note that a recombinant plasmid pET28a-PP13138R was successfully constructed (**Figure 8A**), resulting in the formation of a novel recombinant protein PP13138R with a molecular weight of 98.57 kDa (**Figure 8B**). Our ELISPOT assay demonstrated that the PP13138R vaccine elicited a greater frequency of IFN-γ^+^ T lymphocytes in HCs (**Figure 9A**), ATB patients (**Figure 9B**), and LTBI individuals (**Figure 9C**) when compared to the negative control AIM-V medium. The most significant increase in the number of IFN-γ^+^ T lymphocytes was observed in LTBI subjects (*P* = 0.0236). Moreover, we conducted an *in vitro* evaluation of the PP13138R vaccine’s efficacy in inducing the production of key cytokines, including IFN-γ, IL-2, IL-4, IL-6, IL-10, TNF-α, and IL-17A in HCs, LTBI individuals, and ATB patients. Our analysis revealed that the PP13138R vaccine demonstrated a significant ability to induce the production of specific cytokines upon *in vitro* exposure to PBMCs derived from HCs, LTBI individuals, and ATB patients. The results showed that: (1) the levels of TNF-α produced by PBMCs from HCs (*P* < 0.0001) and ATB patients (*P* = 0.0016) stimulated by PP13138R were significantly higher than those of TNF-α produced by PBMCs from HCs that had been induced with PBS (**Figure 10A**); (2) PBMCs derived from HCs showed a significantly higher level of IFN-γ production when stimulated with PP13138R than those from the LTBI population (*P* = 0.0212) and HCs induced by PBS (*P* = 0.0013, **Figure 10B**); (3) PP13138R elicited significantly higher levels of IL-6 and IL-10 production in PBMCs from HCs (*P* < 0.0001 and *P* = 0.0487), ATB patients (*P* < 0.0001 and *P* = 0.0145), and LTBI individuals (*P* < 0.0001 and *P* < 0.0001) compared to PBMCs from HCs that had been induced with PBS (**Figures 10C, D**). It is noted that the levels of IL-2, IL-4, and IL-17 did not demonstrate any significant differences among the groups in this study (data were not shown).

### 3.7 Principal component analysis and correlation analysis of cytokines induced by the PP13138R vaccine

**Figure 11:**
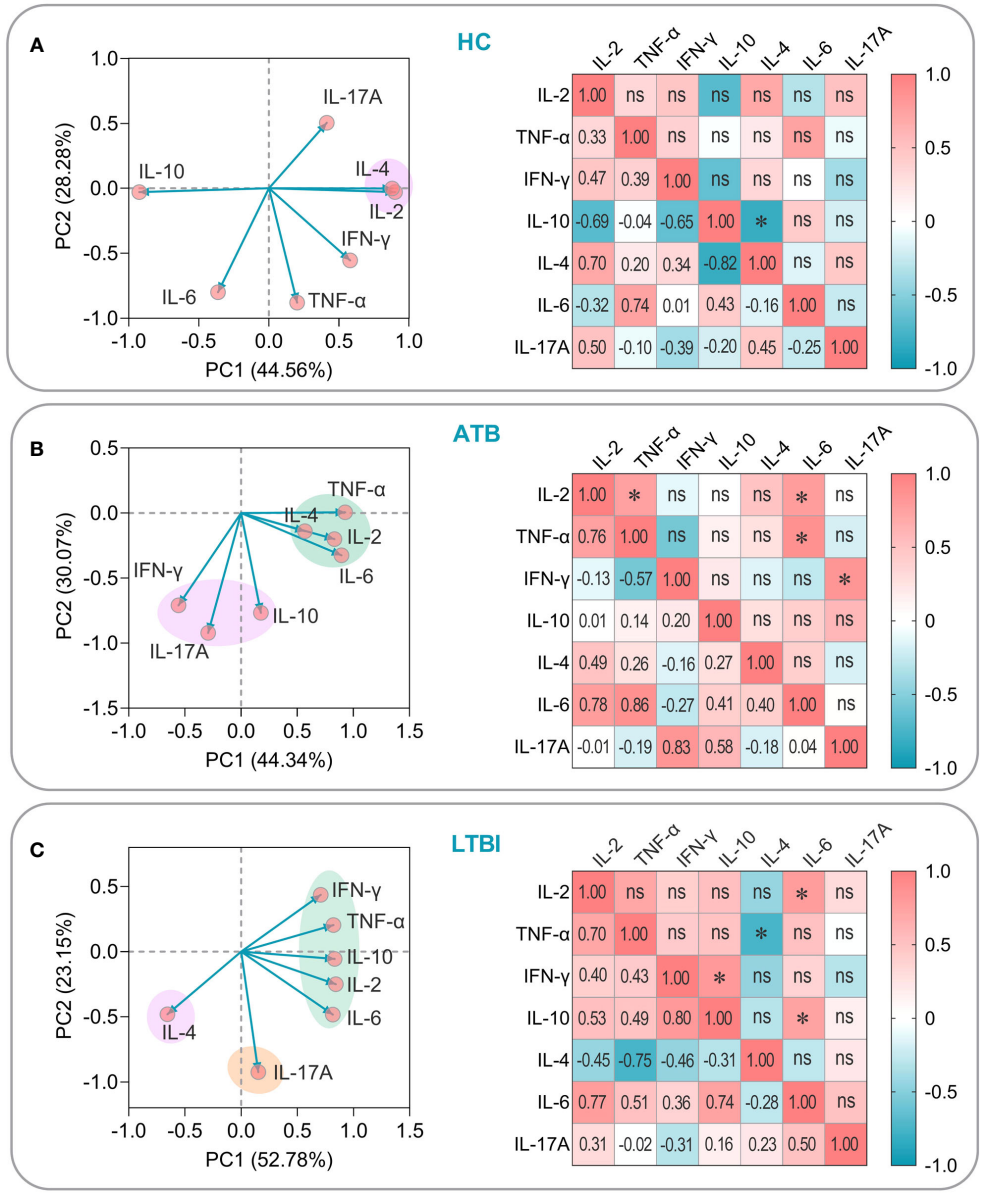
PCA and correlation analysis of cytokines induced by the PP13138R vaccine in HCs, ATB patients, and LTBI populations. The potential relationships among IL-2, TNF-α, IFN-γ, IL-4, IL-6, IL-10, and IL-17A cytokines induced by the PP13138R vaccine in HCs **(A)**, ATB patients **(B)**, and LTBI populations **(C)** were analyzed by using PCA and Pearson’s R analysis using GraphPad Prism 9.5.1 software, respectively. In the PCA analysis, we selected PCs with eigenvalues greater than 1 for further analysis. The two principal components with the largest variance values are shown in the PCA loading plot, and their percentages are shown on the x-and y-axes, respectively. Furthermore, the correlation coefficients R and *P* values of the cytokines with each other were shown in the lower left and upper right halves of the correlation matrix plot, respectively. *P* < 0.05 was considered to be a significant difference. *, *P* < 0.05; ns, no significance.

To establish the potential interrelationship between assorted cytokines present in HCs, individuals afflicted with ATB, and those living with LTBI, we conducted a meticulous PCA and correlation analysis. The resulting findings are as follows: (1) Among HCs, the cumulative variance percentage of the seven cytokines on PC1 and PC2 was 44.56% and 28.28%, respectively. Notably, IL2 and IL-4 were observed to be clustered together and showed a significant positive correlation (*P*=0.024, **Figure 11A**); (2) We discovered that in patients with ATB, IL2, IL-4, IL-6, and TNF-α were clustered together, along with IL-10, IL-17A, and IFN-γ which were clustered into a separate group. Furthermore, correlation analysis uncovered a significant positive correlation between IL-6, IL-2 (*P*=0.040), and TNF-α (*P*=0.013), as well as IFN-γ and IL-17A (*P*=0.022), and IL-2 and TNF-α (*P*=0.049) (**Figure 11B**); (3) Within individuals with LTBI, we noted that IL2, IL-6, IL-10, IFN-γ, and TNF-α were clustered together, whereas IL-4 or IL-17A were arranged into two separate groups. Correlation analysis indicated that there was a significant positive correlation between IL-6 and IL-2 (*P*=0.024) or IL-10 (*P*=0.037). Similarly, our results further revealed a consequentially significant positive correlation between IL-10 and IFN-γ (*P*=0.018), although, interestingly, IL-4 and TNF-α exhibited a significant negative correlation (*P*=0.031) (**Figure 11C**). Our comprehensive analysis of cytokine relationships in the three study groups provides valuable insights into evaluating the immunogenicity and antigenicity of the PP13138R vaccine.

### 3.8 Linear regression analysis of cytokines induced by the PP13138R vaccine

**Figure 12:**
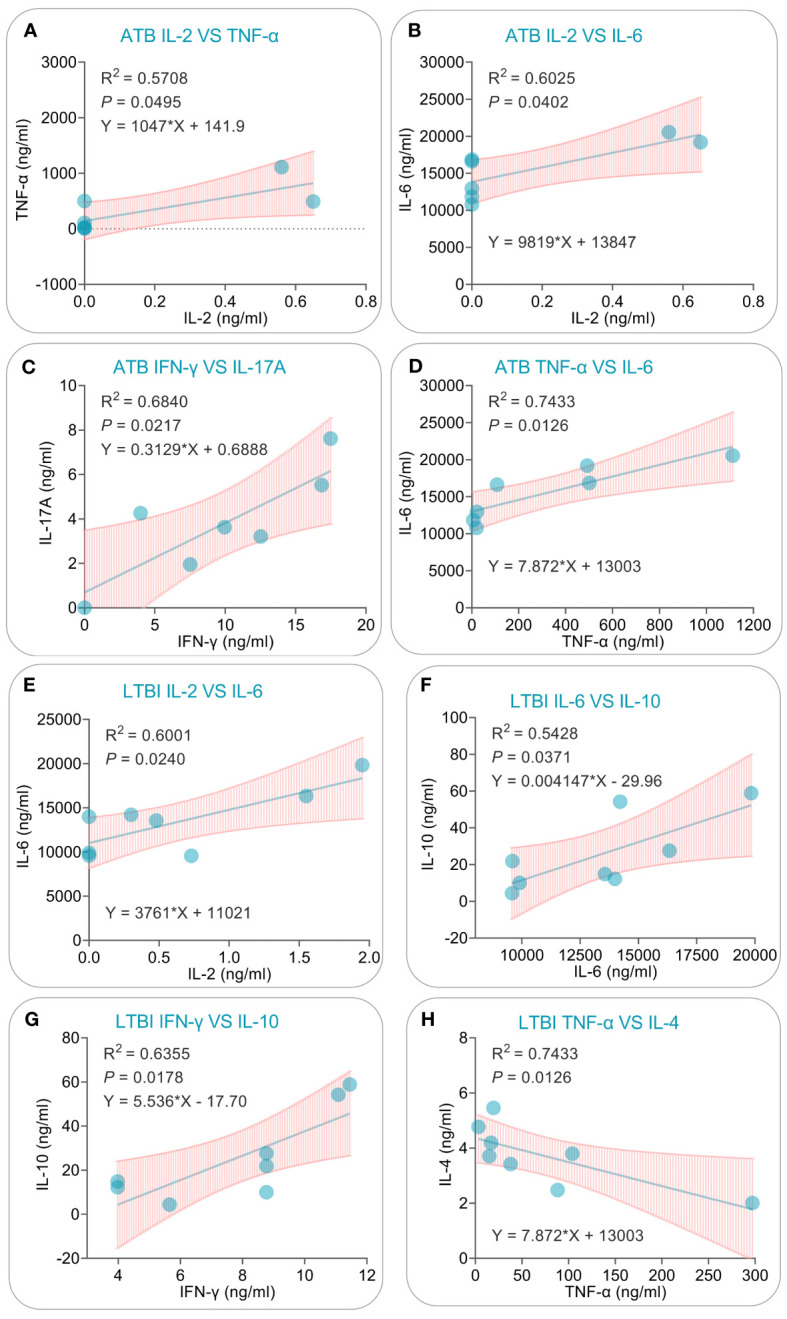
Simple linear regression analysis of cytokines induced by the PP13138R vaccine in ATB patients and individuals with LTBI. The simple linear regression was conducted to evaluate the potential correlations for IL-2 vs. TNF-α **(A)**, IL-2 vs. IL-6 **(B)**, IFN-γ vs. IL-17A **(C)**, TNF-α vs. IL-6 **(D)** in ATB patients, and IL-2 vs. IL-6 **(E)**, IL-6 vs. IL-10 **(F)**, IFN-γ vs. IL-10 **(G)**, and TNF-α vs. IL-4 **(H)** in individuals with LTBI using the GraphPad Prism 9.5.1 software. The R-squared value, *P* value, and equation were displayed in each panel. *P*<0.05 is considered significant.

After conducting PCA and correlation analysis on cytokines produced in response to the PP13138R vaccine, we performed a linear regression analysis of cytokines exhibiting significant correlations in ATB patients and LTBI individuals. In ATB patients, we successfully developed four linear models that exhibited positive correlations between various cytokine combinations. Specifically, the models demonstrated correlations between IL-2 and TNF-α (R2 = 0.5708, *P* = 0.0495, Y = 1047X + 141.9, **Figure 12A**), IL-2 and IL-6 (R2 = 0.6025, *P* = 0.0402, Y = 9819X + 13847, **Figure 12B**), IFN-γ and IL-17A (R2 = 0.6840, *P* = 0.0217, Y = 0.3129X + 0.68888, **Figure 12C**), and TNF-α and IL-6 (R2 = 0.7433, *P* = 0.0126, Y = 7.872X + 13003, **Figure 12D**). Similarly, we were also able to construct three linear models illustrating positive correlations between various cytokines in LTBI subjects. These models included IL-2 and IL-6 (R2 = 0.6001, *P* = 0.0240, Y = 3761X + 11021, **Figure 12E**), IL-6 and IL-10 (R2 = 0.5428, *P* = 0.0371, Y = 0.004147X - 29.96, **Figure 12F**), and IFN-γ and IL-10 (R2 = 0.6355, *P* = 0.0178, Y = 5.536X - 17.70, **Figure 12G**). Additionally, we observed one negatively correlated linear model between TNF-α and IL-4 (R2 = 0.7433, *P* = 0.0126, Y = 7.872X + 13003, **Figure 12H**).

## 4 Discussion

Development of traditional vaccines has historically been a lengthy, costly, and intricate undertaking, exemplified by the 13 years and over 230 culture generations required for the production of BCG, a live attenuated vaccine (45). Nonetheless, the steadily growing field of bioinformatics and immunoinformatics has enabled the rise of online servers and predictive analysis software, thereby giving birth to reverse vaccinology, which has greatly accelerated vaccine design, production, and evaluation processes (46). Thanks to the wide availability of genomes, proteomes, transcriptomes, data analysis programs, and machine learning tools, vaccinology has transitioned into a new phase known as “Reverse Vaccinology 2.0” (47). In a notable trend, Goodswen SJ and colleagues recently released the first guidelines for reverse vaccinology studies that are based on bioinformatics and immunoinformatics techniques in FEMS Microbiology Review in March 2023 (27), which bodes well for the continued advancement of this promising field.

A prior investigation developed a novel MEV to address MTBinfections utilizing 13 HTL epitopes, 13 CTL epitopes, 8 B-cell epitopes, and TLR2 agonist PSMα4. The MEV was named HP13138PB and was shown to have substantial immunogenicity through *in silico* and *in vitro* experiments (20). Moreover, our team developed a new MEV for LTBI named PP19128R, which, in addition to TLR2 agonist PorB, also incorporated the TLR4 agonist RS-09, consequently intensifying immunogenicity and enhancing targeting capabilities (17). A comparative analysis of the experimental outcomes of HP13138PB and PP19128R vaccines indicated superior immunogenicity and enhanced targeting capabilities with vaccines containing both HTL, CTL, and B-cell epitopes along with TLR2 and TLR4 agonists. These results provide valuable insights into the possibility of creating an MEV that, through its incorporation of HTL, CTL, and B cell epitopes and TLR2 and TLR4 agonists, can potentially prevent MTB infection.

In our study on developing an MEV against MTB, selecting potential protective antigens was deemed crucial in ensuring the vaccine’s immunogenicity and efficacy. The MTB H37Rv strain has a large number of genes, 4173 to be precise, which poses a significant challenge in selecting potential candidates for the vaccine’s design (https://mycobrowser.epfl.ch/). With this in mind, previous studies employed various strategies, including data mining and bioinformatics analysis, to narrow down the list of potential candidates. For instance, Anat Zvi and colleagues identified 45 top-hit antigens from among the 3989 open reading frame products of the MTB H37Rv strain (37). In our study, we went a step further and carefully selected 19 antigens from the larger pool based on prior evaluations in either clinical trials or animal models. Notably, the selection criteria also considered the potential for good immunogenicity, antigenicity, and protective efficacy of the antigens. We then screened 13 HTL, 13 CTL, and 8 B-cell immunodominant epitopes from these 19 antigens to construct a novel MEV, named PP13138R, designed to prevent MTB infection. The study’s approach, which aligned with the recommended four-step process for classical MEV design (27), embraced the importance of selecting protective antigens carefully to enhance MEV efficiency. Notably, the approach utilized in this study could have implications in developing vaccines for pathogens with a large number of possible vaccine targets.

Accurate recognition of vaccines by antigen-presenting cells (APCs) and their efficient presentation to adaptive immune cells is critical to activating immune responses. Therefore, MHC I/II binding score, immunogenicity, antigenicity, and IFN-γ inducing ability are key components that require optimal screening during the screening process of immunodominant epitopes (48–50). Herein, to ensure the PP13138R vaccine’s accuracy and effectiveness, we employed a rigorous evaluation process that involved analyzing the vaccine’s structure and properties, including the construction of the secondary, tertiary, tertiary structure, and recombinant plasmid. This approach ensured the addition of an evaluation step at each construction stage, as recommended in the optimal screening principles (27). Based on the evaluation results, the PP13138R vaccine demonstrated promising immunogenicity, antigenicity, stability, and IFN-γ inducing ability without any signs of toxicity and sensitization. These findings suggest that PP13138R is promising as a vaccine to prevent MTB infection.

It has been reported that TLR is one of the most classical pattern recognition receptors for immune cells to recognize MTB (51, 52). In particular, TLR2 and TLR4 and their ligand MyD88 are most important in initiating the innate immune response to eliminate and kill MTB (51, 53, 54). This study included TLR2 and TLR4 agonists and adjuvant peptides in the PP13138R vaccine to enhance vaccine immunogenicity and targeting. We analyzed the binding ability of the PP13138R vaccine to TLR2 and TLR4 and found that this vaccine can tightly bind to both TLR2 and TLR4. This result indicated that the PP13138R vaccine theoretically could accurately initiate innate immune responses. Furthermore, we found that after stimulation with the PP13138R vaccine, the number of IFN-γ^+^ T lymphocytes in HCs, ATB patients, and LTBI individuals was higher than that in three groups stimulated with AIM-V medium. Especially, the number of IFN-γ^+^ T lymphocytes in LTBI individuals rather than ATB patients was significantly higher. Contrary to our results, another study found that CD4^+^IFN-γ^+^ T lymphocytes in ATB patients were substantially higher than in LTBI and HCs (55). This difference may stem from differences in the antigen and sample size used in the two studies.

In addition, we also observed that the PP13138R vaccine could stimulate PBMCs from HCs, ATB patients, or LTBI individuals to secrete significantly higher levels of IFN-γ, TNF-α, IL-6, and IL-10 *in vitro* compared to PBS. Previous studies have demonstrated that IFN-γ^+^ T lymphocytes can kill MTB by secreting high levels of IFN-γ to induce Th1-type T cell proliferation and promote CTL to secrete substances such as perforin and granzyme (42, 43, 56, 57). Interestingly, we found that PP13138R induced high levels of both the pro-inflammatory factor IFN-γ, IL-2, IL-6 and the anti-inflammatory factor IL-10 in PBMC obtained from ATB patients or LTBI individuals. This finding may initially seem contradictory since IL-10 is typically associated with dampening inflammation, while IFN-γ, IL-2, and IL-6 are known to promote inflammatory responses. However, this positive correlation can be explained by the complex and dynamic interplay between different components of the immune system during MTB infection. Previous studies have demonstrated that MTB infection relies on a balance of pro-inflammatory and anti-inflammatory mediators to regulate the formation of granulomas (58). In addition, besides its anti-inflammatory role, IL-10 also acts as an immunomodulatory cytokine that regulates and shapes immune responses (59, 60). IL-10 can suppress excessive inflammation, but it can also promote immune activation and proliferation of T cells, including the production of IFN-γ, IL-2, and IL-6 (10, 61). It is also worth noting that the immune responses in individuals with ATB and LTBI can exhibit variations due to differences in the immune status and disease progression (3, 10). These differences may contribute to the observed variations in cytokine correlations. However, further investigation is needed to fully elucidate the underlying mechanisms.

Furthermore, although the PP13138R vaccine could induce more IFN-γ^+^ T lymphocytes in LTBI individuals, it did not induce significantly higher IFN-γ production in PBMCs from LTBI individuals *in vitro* than in negative controls. These data suggest that the number of IFN-γ^+^ T lymphocytes induced by the vaccine is not necessarily proportional to the concentration of the cytokine IFN-γ detected *in vitro*. Therefore, more indicators should be introduced to evaluate vaccine immunogenicities, such as lymphocyte subsets, specific biomarkers, high-throughput cytokine assays, and transcriptome sequencing.

Furthermore, although the PP13138R vaccine could induce more IFN-γ^+^ T lymphocytes in individuals with LTBI, it did not result in a significantly higher IFN-γ production in PBMCs from LTBI individuals *in vitro* compared to negative controls. These findings suggest that the number of IFN-γ^+^ T lymphocytes induced by the vaccine may not directly correlate with the concentration of the cytokine IFN-γ detected *in vitro*. It is important to consider that IFN-γ can be produced by various cell types, including CD4^+^ and CD8^+^ T lymphocytes, as well as NK cells (16, 31, 61). In our assays, we focused on the measurement of IFN-γ production by PBMCs as a whole, which includes a mixture of different immune cell populations. Therefore, the observed levels of IFN-γ might not solely reflect the contribution of T lymphocytes. To gain a more comprehensive understanding of vaccine immunogenicity, it would be beneficial to incorporate additional indicators and assessments. These could include analyzing specific lymphocyte subsets, investigating the expression of relevant biomarkers, employing high-throughput cytokine assays to evaluate a broader cytokine profile, and performing transcriptome sequencing analysis to explore gene expression changes induced by the vaccine. These approaches would allow us to capture a more detailed and multidimensional view of the immune response induced by the PP13138R vaccine in LTBI individuals.

The study’s findings are subject to several limitations to be considered in interpreting the results. First, the spatial structure and interactions of the PP13138R vaccine with TLR2 and TLR4 were predicted using bioinformatic techniques without validation through experimentation. Although computational predictions are useful for guiding experiments and driving research, variations between predicted and actual molecular configurations may limit the accuracy of the described vaccine design. Second, while thorough immune analyses of the PP13138R vaccine were conducted *in silico* and *in vitro*, only a limited subset of cytokines and IFN-γ^+^ T lymphocytes were evaluated experimentally. The significance of such responses toward protecting against infection remains partially unknown. Third, although *in silico* simulations using C-ImmSim suggested that innate and adaptive immune cells can produce various cytokines post-vaccine stimulation, only seven major Th1/Th2/Th17 cytokines were validated experimentally. Fourth, the protective efficacy of the PP13138R vaccine has not been assessed in an animal model, which is essential for the pre-clinical evaluation of vaccine candidates. As a result, it remains to be determined whether the PP13138R vaccine can generate protective cellular and cytokine responses similar to that hinted by the computational modeling and experimental data presented herein.

## 5 Conclusion

This study, PP13138R, developed a novel MEV against MTB infection. The vaccine comprises 13 HTL epitopes, 13 CTL epitopes, and 8 B-cell epitopes, along with TLR-2 and TLR-4 agonists PorB and RS-09 and the helper peptide PADRE. Bioinformatics analysis demonstrated that the vaccine had good antigenicity and immunogenicity while being non-toxic and non-sensitizing. Additionally, immunoinformatics analysis indicated strong binding between the PP13138R vaccine and TLR2/TLR4, which could lead to innate and adaptive immune responses. *In vitro* experiments confirmed that the vaccine elicited IFN-γ^+^ T lymphocytes and produced cytokines such as IL-10, IFN-γ, IL-6, and TNF-α in individuals with different TB conditions such as HCs, ATB patients, and LTBI individuals. These findings illustrate the potential of reverse vaccinology in designing preventive TB vaccines and that PP13138R offers a promising candidate for preventing MTB infection.

## Data availability statement

The original contributions presented in the study are included in the article/**Supplementary Files**, further inquiries can be directed to the corresponding author/s.

## Ethics statement

The studies involving humans were approved by Ethics Committee of the Eighth Medical Center of PLA General Hospital (Approval No: 309202204080808). The studies were conducted in accordance with the local legislation and institutional requirements. The participants provided their written informed consent to participate in this study.

## Author contributions

FJ: Data curation, Formal Analysis, Methodology, Software, Visualization, Writing – original draft. YH: Data curation, Formal Analysis, Methodology, Writing – original draft. YL: Methodology, Writing – review & editing. YX: Methodology, Writing – review & editing. PC: Methodology, Software, Visualization, Writing – review & editing. LX: Conceptualization, Resources, Writing – review & editing. WG: Conceptualization, Funding acquisition, Project administration, Resources, Supervision, Visualization, Writing – review & editing.

## Supplementary material

Supplementary Figure 1 Flow chart of the PP13138R vaccine design and evaluation.

Supplementary materials S1 Details regarding *in silico* immunization scheme.

## Glossary

**Table:**

ATB	active tuberculosis
BCG	bacillus Calmette-Guérin
CTL	cytotoxic T lymphocytes
ELISPOT	enzyme-linked immunospot assay
GRAVY	grand average of hydropathicity
HTL	helper T lymphocytes
HCs	healthy controls
IL-2	interleukin-2
IFN-γ	interferon gamma
IEDB	immune epitope database
LTBI	latent tuberculosis infection
MTB	*Mycobacterium tuberculosis*
NHFPC	National Health and Family Planning Commission of China
PBMCs	peripheral blood mononuclear cells
SEM	standard error of the mean
TLR	toll-like receptor
TB	tuberculosis
TNF-α	tumor necrosis factor
WHO	World Health Organization
MEV	Multi-epitope vaccine
PTB	pulmonary tuberculosis
LMICs	low- and middle-income countries
HLA-I	human leukocyte antigen I
IGRA	interferon-gamma release test
ESAT-6	EC test, recombinant MTB fusion protein of early secretory antigenic target protein 6-kDa
CFP-10	culture filtrate protein 10 kDa
HIV	human immunodeficiency virus
MHC II	major histocompatibility complex II
PDB	protein data bank
MMDB	Molecular Modeling Database
*E. coli*	*Escherichia coli*
LB	Luria-Bertani
SDS-PAGE	sodium dodecyl sulfate-polyacrylamide gel electrophoresis
IFN-γ+	interferon-gamma positive
PBS	phosphate-buffered saline
TNF-α	tumor necrosis factor-α
CBA	Cytometric Bead Assay
SFCs	spot-forming cells
PCA	principal component analysis
DCs	dendritic cells
Th	helper T
ANOVA	one-way analysis of variance.
