# Supplementary figures and images for: A comprehensive approach to developing a multi-epitope vaccine against Mycobacterium tuberculosis: from in silico design to in vitro immunization evaluation
Source: Front Immunol. 2023 Nov 2;14:1280299. doi: 10.3389/fimmu.2023.1280299 (PMC10652892; doi:10.3389/fimmu.2023.1280299)

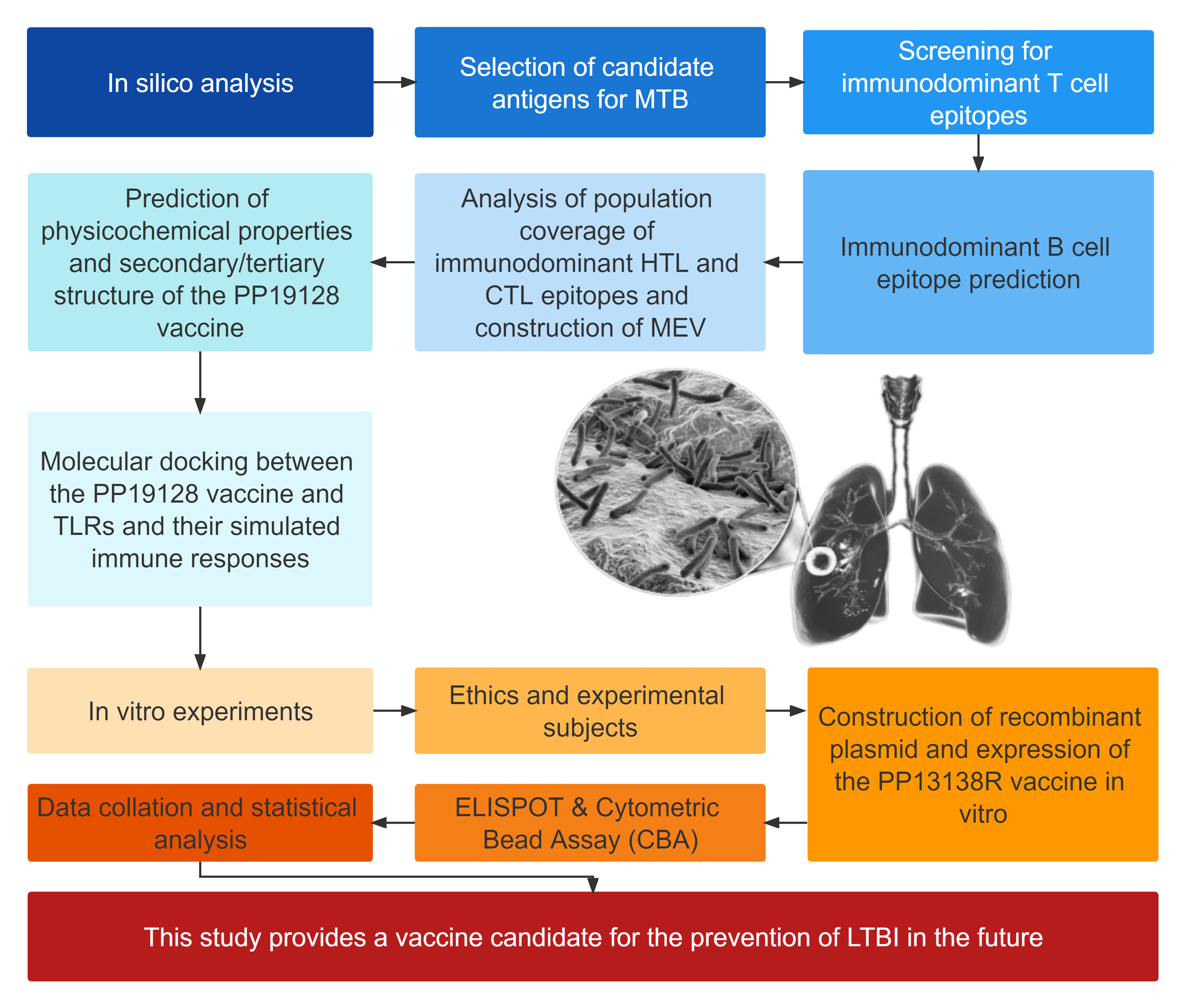

Supplement: Supplementary Figure 1 — Flow chart of the PP13138R vaccine design and evaluation. [file Image_1.png]
