# Supplementary material for: A comprehensive approach to developing a multi-epitope vaccine against Mycobacterium tuberculosis: from in silico design to in vitro immunization evaluation
Source: Front Immunol. 2023 Nov 2;14:1280299. doi: 10.3389/fimmu.2023.1280299 (PMC10652892; doi:10.3389/fimmu.2023.1280299)
Supplement: Supplementary materials S1 — Details regarding in silico immunization scheme. [file Presentation_1.pdf]

C-IMMSIM simulation results

February 7, 2023

Abstract

This document includes the plots relative to the simulation and the outcome of the epitope/peptide prediction used.

Produced by the C-IMMSIM Online server available at `http://kraken.iac.rm.cnr.it/C-IMMSIM`

CITATIONS: For publication of results, please cite:

Nicolas Rapin, Ole Lund, Massimo Bernaschi, Filippo Castiglione. Computational Immunology Meets Bioinformatics: The Use of Prediction Tools for Molecular Binding in the Simulation of the Immune System. PLoS ONE 5(4): e9862. doi:10.1371/journal.pone.0009862, 2010.

Original C-IMMSIM model: `www.iac.cnr.it/∼filippo/c-immsim`

GETTING HELP: Scientific problems: Filippo Castiglione (f dot castiglione at iac dot cnr dot it) Technical problems: Ilaria Gonnella (ilaria dot gonnella at cnr dot it)

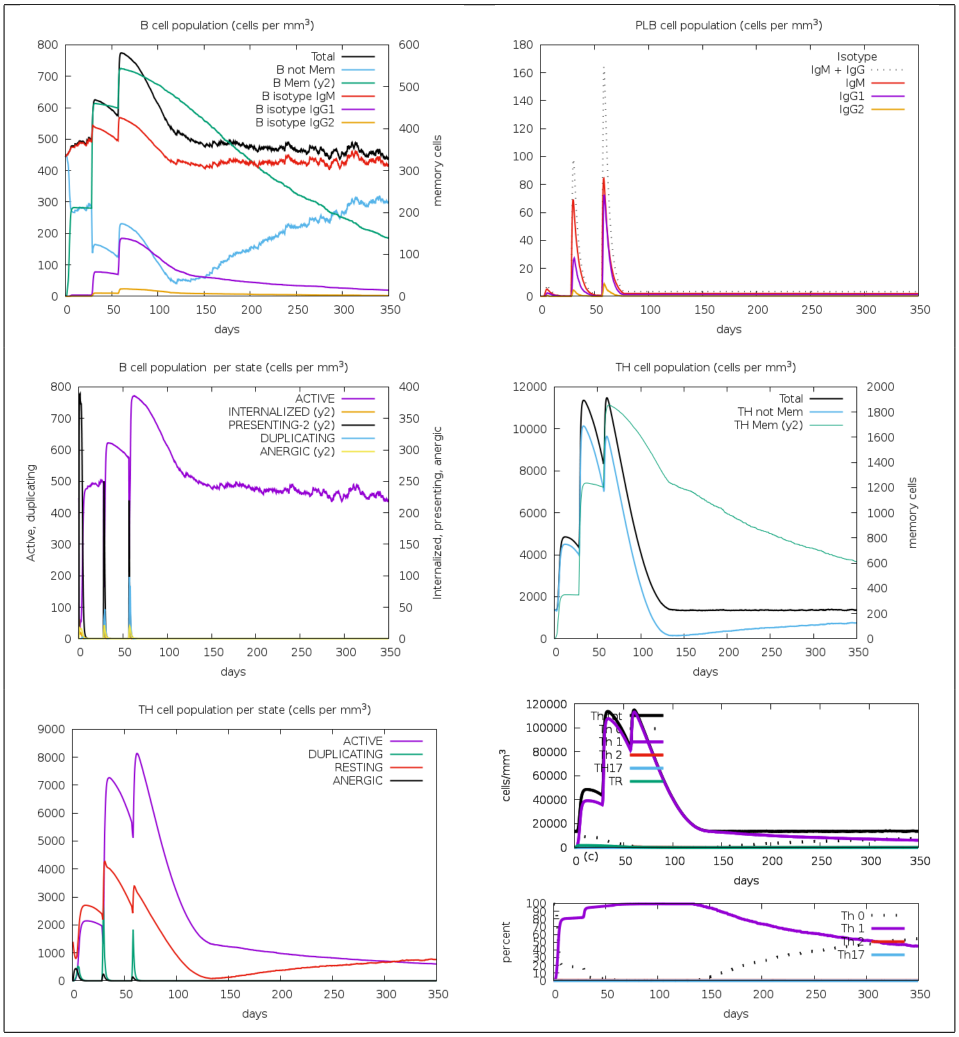

Figure 1: Cell counts shown. Legend: Act=active, Intern=internalized the Ag, Pres II = presenting on MHC II, Dup = in the mitotic cycle, Anergic = anergic, Resting = not active.

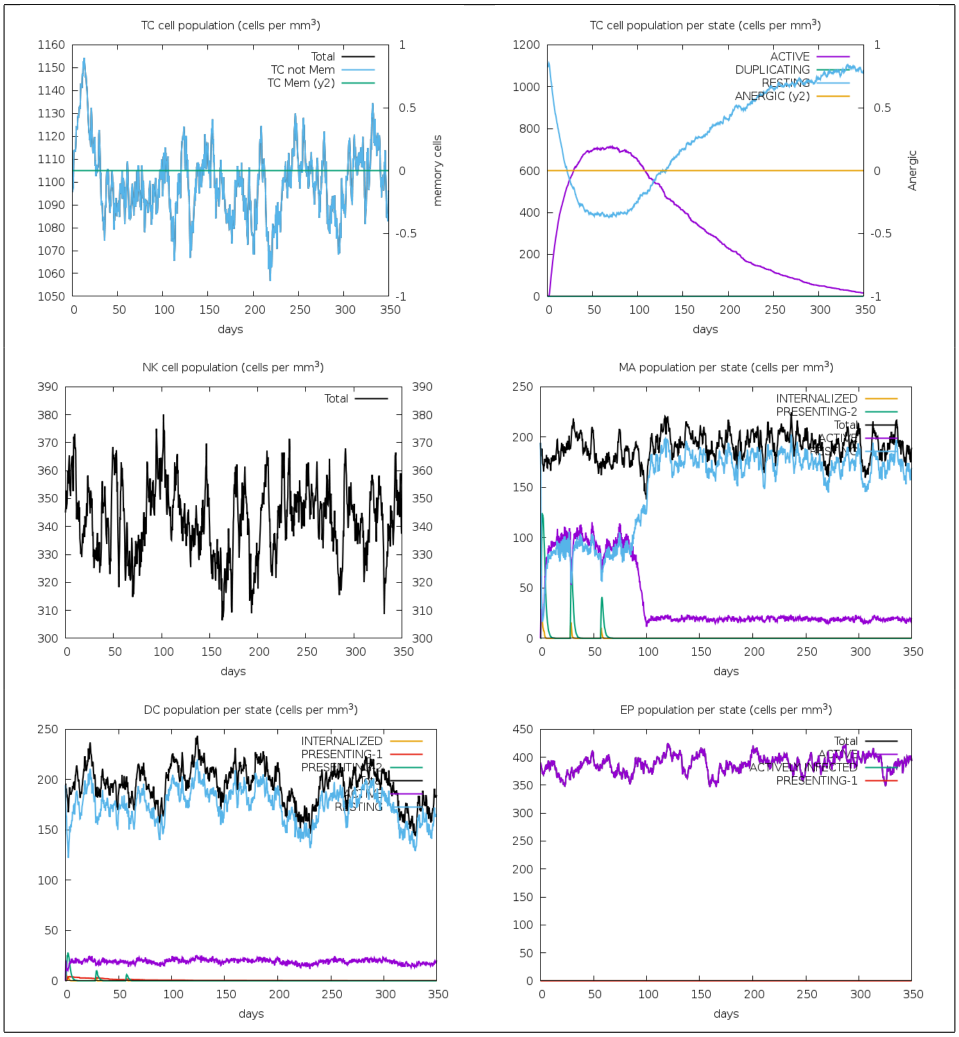

Figure 2: Legend: symbols as figure above.

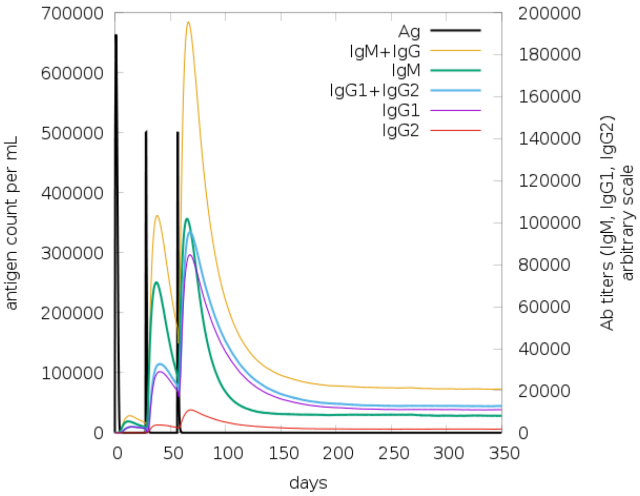

Figure 3: The virus, the immunoglobulins and the immunocomplexes.

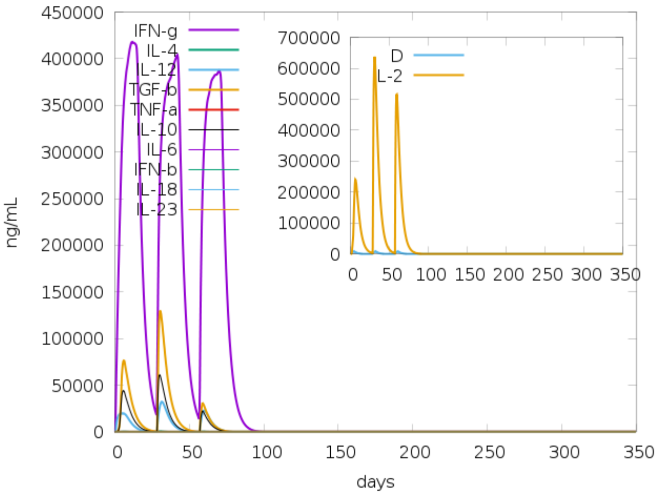

Figure 4: Concentration of cytokines and interleukins. Inset plot shows danger signal together with leukocyte growth factor IL-2.

```
Parker_B_scale_epitope_find
Use Parker's propensity scale, takes an antigen block as input,
and creates a list of residues that are possible epitopes.


----------------------------------
Antigen sequence file: /opt/lampp/htdocs/C-IMMSIM/Jobs/input/9107_20230207-091852_5_5fvJbmMTm4C6.FSA_1_001
----------------------------------


IALTLAALPVAAMADVTLYGTIKAGVETSRSVAHNGAQAASVETGTGIVDLGSKIGFKGQEDLGNGLKAIWQVEQEAAAK
AGLFQRHGEGTKATVGEPVGPGPGTGSAVVGLSMAASSALTLGPGPGAAIGLSMAGSSAMILAAGPGPGTNIRQAGVQYS
RAGPGPGAQGGYGAMALAAFHGPGPGMSDPAYNINISLPSYYPDGPGPGALSAGVGAVAMSLTVGAGPGPGRRRSLRWSW
LLSVLAAGPGPGAELTAAQVRVAAAAYETAGPGPGYGYAAASAVAARLNPLGPGPGGTVGGAAPGGGAAAAVAHGPGPGV
APSVMPAAAAGSSATGPGPGTGAVLGGGGIAMAAGPGPGLLLVLAFAGGYAVAACAAYGSAVVGLSMAAYNAAGGHNAVA
AYTAAQAAVVRAAYAMSGDIVGAAAYRTADGINYRAAYAAIGGGVAVAAYATATATATLAAYFLNLDVPLFVAAYQAETA
VNTLAAYAVNGEPAPLAAYGLSNATPREVAAYQVTRNRIKKAAYNLPPPLAPDFKKAKFLEGFVRTSNIKFQDAYNKKAP
DDPICTGGGNIMAHVSYVKKAGLSASSGLAHADSLASSASKKIGQAAEAMGNSRMFYNQYRSKKALGQTVQASDSLTGAE
ETLNKKVNTLFEKLEPMASILDPGASKKAVGERVLATQGRGAWPVCGRKKAYHAMSSTHEANTMAMMARDEAAAKAPPHA
LSHHHHHH

00000000000000000000000001110100000001000011000000000000001011100000000000101000
00000001111101000010001110000000000000000000000000000000000000000011000000000000
00110111010000000000000001100000000000000000111000000000000000000001111100000000
00000000001000000000000000000111010000000000000000000010111000110000000000000000
00000000000111111111000000000000000000000000000000000000000000000000000001000000
00000000000000000000000000010100000000000000000000000000000000000000000000000000
00000000000000000000000010000000000000000000000000000000000000000000000000110111
00010000000000000000000000000000000000100000000001100000000111000000011101111000
11100000000000000000000000110011000000001000000000000000001111000000000011100000 00
00000000
Epitopes of protein 0 ------------
1]       pos=87 len=5        GEGTK
2]       pos=227 len=5        PGPGR
3]       pos=331 len=9        SSATGPGPG
4]       pos=633 len=4        DSLT
5]       pos=696 len=4        STHE
```

```
DoPeptideList_I:
        Given the antigen injected creates the list of peptides for all the
        NumAgProts proteins and for all i.e., 4 MHCI molecules

        Read class I peptide list from file? NO

========================================================================

Allele: A0101
Pseudo sequence: KAVHAEQRNKAQTRA
Threshold: 9.456400
Max score: 29.236000


----------------------------------
Antigen sequence file: /opt/lampp/htdocs/C-IMMSIM/Jobs/input/9107_20230207-091852_5_5fvJbmMTm4C6.FSA_1_001
----------------------------------

IALTLAALPVAAMADVTLYGTIKAGVETSRSVAHNGAQAASVETGTGIVDLGSKIGFKGQEDLGNGLKAIWQVEQEAAAK
AGLFQRHGEGTKATVGEPVGPGPGTGSAVVGLSMAASSALTLGPGPGAAIGLSMAGSSAMILAAGPGPGTNIRQAGVQYS
```

```
RAGPGPGAQGGYGAMALAAFHGPGPGMSDPAYNINISLPSYYPDGPGPGALSAGVGAVAMSLTVGAGPGPGRRRSLRWSW
LLSVLAAGPGPGAELTAAQVRVAAAAYETAGPGPGYGYAAASAVAARLNPLGPGPGGTVGGAAPGGGAAAAVAHGPGPGV
APSVMPAAAAGSSATGPGPGTGAVLGGGGIAMAAGPGPGLLLVLAFAGGYAVAACAAYGSAVVGLSMAAYNAAGGHNAVA
AYTAAQAAVVRAAYAMSGDIVGAAAYRTADGINYRAAYAAIGGGVAVAAYATATATATLAAYFLNLDVPLFVAAYQAETA
VNTLAAYAVNGEPAPLAAYGLSNATPREVAAYQVTRNRIKKAAYNLPPPLAPDFKKAKFLEGFVRTSNIKFQDAYNKKAP
DDPICTGGGNIMAHVSYVKKAGLSASSGLAHADSLASSASKKIGQAAEAMGNSRMFYNQYRSKKALGQTVQASDSLTGAE
ETLNKKVNTLFEKLEPMASILDPGASKKAVGERVLATQGRGAWPVCGRKKAYHAMSSTHEANTMAMMARDEAAAKAPPHA
LSHHHHHH

Epitopes of protein 0 ------------
0]        pos= 453 score=0.000978 unnormalised=0.1026000000        TATATLAAY
1]        pos=  -1 score=0.999022 unnormalised=104.8410000000        non-binding event

==========================================================================

Allele: A0201
Pseudo sequence: KAAHVEQRKAQTRTV
Threshold: 9.523800
Max score: 27.437000


----------------------------------
Antigen sequence file: /opt/lampp/htdocs/C-IMMSIM/Jobs/input/9107_20230207-091852_5_5fvJbmMTm4C6.FSA_1_001
----------------------------------

IALTLAALPVAAMADVTLYGTIKAGVETSRSVAHNGAQAASVETGTGIVDLGSKIGFKGQEDLGNGLKAIWQVEQEAAAK
AGLFQRHGEGTKATVGEPVGPGPGTGSAVVGLSMAASSALTLGPGPGAAIGLSMAGSSAMILAAGPGPGTNIRQAGVQYS
RAGPGPGAQGGYGAMALAAFHGPGPGMSDPAYNINISLPSYYPDGPGPGALSAGVGAVAMSLTVGAGPGPGRRRSLRWSW
LLSVLAAGPGPGAELTAAQVRVAAAAYETAGPGPGYGYAAASAVAARLNPLGPGPGGTVGGAAPGGGAAAAVAHGPGPGV
APSVMPAAAAGSSATGPGPGTGAVLGGGGIAMAAGPGPGLLLVLAFAGGYAVAACAAYGSAVVGLSMAAYNAAGGHNAVA
AYTAAQAAVVRAAYAMSGDIVGAAAYRTADGINYRAAYAAIGGGVAVAAYATATATATLAAYFLNLDVPLFVAAYQAETA
VNTLAAYAVNGEPAPLAAYGLSNATPREVAAYQVTRNRIKKAAYNLPPPLAPDFKKAKFLEGFVRTSNIKFQDAYNKKAP
DDPICTGGGNIMAHVSYVKKAGLSASSGLAHADSLASSASKKIGQAAEAMGNSRMFYNQYRSKKALGQTVQASDSLTGAE
ETLNKKVNTLFEKLEPMASILDPGASKKAVGERVLATQGRGAWPVCGRKKAYHAMSSTHEANTMAMMARDEAAAKAPPHA
LSHHHHHH

Epitopes of protein 0 ------------
0]        pos= 132 score=0.006134 unnormalised=0.6862000000        SMAGSSAMI
1]        pos= 185 score=0.005848 unnormalised=0.6542000000        GMSDPAYNI
2]        pos= 209 score=0.008833 unnormalised=0.9882000000        ALSAGVGAV
3]        pos= 457 score=0.019507 unnormalised=2.1822000000        TLAAYFLNL
4]        pos= 569 score=0.019605 unnormalised=2.1932000000        NIMAHVSYV
5]        pos= 648 score=0.002907 unnormalised=0.3252000000        TLFEKLEPM
6]        pos=  -1 score=0.937166 unnormalised=104.8410000000        non-binding event

==========================================================================

Allele: B0702
Pseudo sequence: KAAREEQQIKAQTRE
Threshold: 8.702800
Max score: 28.406000


----------------------------------
Antigen sequence file: /opt/lampp/htdocs/C-IMMSIM/Jobs/input/9107_20230207-091852_5_5fvJbmMTm4C6.FSA_1_001
----------------------------------

IALTLAALPVAAMADVTLYGTIKAGVETSRSVAHNGAQAASVETGTGIVDLGSKIGFKGQEDLGNGLKAIWQVEQEAAAK
AGLFQRHGEGTKATVGEPVGPGPGTGSAVVGLSMAASSALTLGPGPGAAIGLSMAGSSAMILAAGPGPGTNIRQAGVQYS
RAGPGPGAQGGYGAMALAAFHGPGPGMSDPAYNINISLPSYYPDGPGPGALSAGVGAVAMSLTVGAGPGPGRRRSLRWSW
LLSVLAAGPGPGAELTAAQVRVAAAAYETAGPGPGYGYAAASAVAARLNPLGPGPGGTVGGAAPGGGAAAAVAHGPGPGV
APSVMPAAAAGSSATGPGPGTGAVLGGGGIAMAAGPGPGLLLVLAFAGGYAVAACAAYGSAVVGLSMAAYNAAGGHNAVA
AYTAAQAAVVRAAYAMSGDIVGAAAYRTADGINYRAAYAAIGGGVAVAAYATATATATLAAYFLNLDVPLFVAAYQAETA
VNTLAAYAVNGEPAPLAAYGLSNATPREVAAYQVTRNRIKKAAYNLPPPLAPDFKKAKFLEGFVRTSNIKFQDAYNKKAP
DDPICTGGGNIMAHVSYVKKAGLSASSGLAHADSLASSASKKIGQAAEAMGNSRMFYNQYRSKKALGQTVQASDSLTGAE
```

```
ETLNKKVNTLFEKLEPMASILDPGASKKAVGERVLATQGRGAWPVCGRKKAYHAMSSTHEANTMAMMARDEAAAKAPPHA
LSHHHHHH

Epitopes of protein 0 ------------
0]        pos=   7 score=0.004079 unnormalised=0.5162000000        LPVAAMADV
1]        pos=  99 score=0.012274 unnormalised=1.5532000000        GPGPGTGSA
2]        pos= 101 score=0.001977 unnormalised=0.2502000000        GPGTGSAVV
3]        pos= 113 score=0.010915 unnormalised=1.3812000000        MAASSALTL
4]        pos= 204 score=0.005549 unnormalised=0.7022000000        GPGPGALSA
5]        pos= 282 score=0.002040 unnormalised=0.2582000000        AVAARLNPL
6]        pos= 302 score=0.026720 unnormalised=3.3812000000        APGGGAAAA
7]        pos= 316 score=0.006252 unnormalised=0.7912000000        GPGVAPSVM
8]        pos= 320 score=0.031019 unnormalised=3.9252000000        APSVMPAAA
9]        pos= 324 score=0.031264 unnormalised=3.9562000000        MPAAAAGSS
10]        pos= 335 score=0.027984 unnormalised=3.5412000000        GPGPGTGAV
11]        pos= 525 score=0.000081 unnormalised=0.0102000000        LPPPLAPDF
12]        pos= 712 score=0.011334 unnormalised=1.4342000000        AAKAPPHAL
13]        pos=  -1 score=0.828510 unnormalised=104.8410000000        non-binding event

=========================================================================

Allele: B0801
Pseudo sequence: KAAREEQQIKAQTRE
Threshold: 8.405400
Max score: 30.890000


----------------------------------
Antigen sequence file: /opt/lampp/htdocs/C-IMMSIM/Jobs/input/9107_20230207-091852_5_5fvJbmMTm4C6.FSA_1_001
----------------------------------

IALTLAALPVAAMADVTLYGTIKAGVETSRSVAHNGAQAASVETGTGIVDLGSKIGFKGQEDLGNGLKAIWQVEQEAAAK
AGLFQRHGEGTKATVGEPVGPGPGTGSAVVGLSMAASSALTLGPGPGAAIGLSMAGSSAMILAAGPGPGTNIRQAGVQYS
RAGPGPGAQGGYGAMALAAFHGPGPGMSDPAYNINISLPSYYPDGPGPGALSAGVGAVAMSLTVGAGPGPGRRRSLRWSW
LLSVLAAGPGPGAELTAAQVRVAAAAYETAGPGPGYGYAAASAVAARLNPLGPGPGGTVGGAAPGGGAAAAVAHGPGPGV
APSVMPAAAAGSSATGPGPGTGAVLGGGGIAMAAGPGPGLLLVLAFAGGYAVAACAAYGSAVVGLSMAAYNAAGGHNAVA
AYTAAQAAVVRAAYAMSGDIVGAAAYRTADGINYRAAYAAIGGGVAVAAYATATATATLAAYFLNLDVPLFVAAYQAETA
VNTLAAYAVNGEPAPLAAYGLSNATPREVAAYQVTRNRIKKAAYNLPPPLAPDFKKAKFLEGFVRTSNIKFQDAYNKKAP
DDPICTGGGNIMAHVSYVKKAGLSASSGLAHADSLASSASKKIGQAAEAMGNSRMFYNQYRSKKALGQTVQASDSLTGAE
ETLNKKVNTLFEKLEPMASILDPGASKKAVGERVLATQGRGAWPVCGRKKAYHAMSSTHEANTMAMMARDEAAAKAPPHA
LSHHHHHH

Epitopes of protein 0 ------------
0]        pos= 282 score=0.003797 unnormalised=0.4096000000        AVAARLNPL
1]        pos= 641 score=0.020703 unnormalised=2.2336000000        TLNKKVNTL
2]        pos= 712 score=0.003732 unnormalised=0.4026000000        AAKAPPHAL
3]        pos=  -1 score=0.971769 unnormalised=104.8410000000        non-binding event
```

```
DoPeptideList_II:
        Given the antigen injected creates the list of peptides for all the
        NumAgProts proteins and for all i.e., 2 MHCII molecules

        Read class II peptide list from file? NO

=========================================================================

Allele: DRB1_0101
Pseudo sequence: KAFAHVEQRKAQTRV
Threshold: 2.392440
Max score: 26.461000
```

```
----------------------------------
Antigen sequence file: /opt/lampp/htdocs/C-IMMSIM/Jobs/input/9107_20230207-091852_5_5fvJbmMTm4C6.FSA_1_001
----------------------------------

IALTLAALPVAAMADVTLYGTIKAGVETSRSVAHNGAQAASVETGTGIVDLGSKIGFKGQEDLGNGLKAIWQVEQEAAAK
AGLFQRHGEGTKATVGEPVGPGPGTGSAVVGLSMAASSALTLGPGPGAAIGLSMAGSSAMILAAGPGPGTNIRQAGVQYS
RAGPGPGAQGGYGAMALAAFHGPGPGMSDPAYNINISLPSYYPDGPGPGALSAGVGAVAMSLTVGAGPGPGRRRSLRWSW
LLSVLAAGPGPGAELTAAQVRVAAAAYETAGPGPGYGYAAASAVAARLNPLGPGPGGTVGGAAPGGGAAAAVAHGPGPGV
APSVMPAAAAGSSATGPGPGTGAVLGGGGIAMAAGPGPGLLLVLAFAGGYAVAACAAYGSAVVGLSMAAYNAAGGHNAVA
AYTAAQAAVVRAAYAMSGDIVGAAAYRTADGINYRAAYAAIGGGVAVAAYATATATATLAAYFLNLDVPLFVAAYQAETA
VNTLAAYAVNGEPAPLAAYGLSNATPREVAAYQVTRNRIKKAAYNLPPPLAPDFKKAKFLEGFVRTSNIKFQDAYNKKAP
DDPICTGGGNIMAHVSYVKKAGLSASSGLAHADSLASSASKKIGQAAEAMGNSRMFYNQYRSKKALGQTVQASDSLTGAE
ETLNKKVNTLFEKLEPMASILDPGASKKAVGERVLATQGRGAWPVCGRKKAYHAMSSTHEANTMAMMARDEAAAKAPPHA
LSHHHHHH

Epitopes of protein 0 ------------
0]        pos=   1 score=0.000194 unnormalised=0.0685600000        ALTLAALPV
1]        pos=   4 score=0.014020 unnormalised=4.9635600000        LAALPVAAM
2]        pos=   9 score=0.005857 unnormalised=2.0735600000        VAAMADVTL
3]        pos=  31 score=0.012947 unnormalised=4.5835600000        VAHNGAQAA
4]        pos=  72 score=0.010433 unnormalised=3.6935600000        VEQEAAAKA
5]        pos= 108 score=0.007058 unnormalised=2.4985600000        VVGLSMAAS
6]        pos= 110 score=0.004304 unnormalised=1.5235600000        GLSMAASSA
7]        pos= 111 score=0.031963 unnormalised=11.3155600000        LSMAASSAL
8]        pos= 113 score=0.016613 unnormalised=5.8815600000        MAASSALTL
9]        pos= 119 score=0.006625 unnormalised=2.3455600000        LTLGPGPGA
10]        pos= 121 score=0.001609 unnormalised=0.5695600000        LGPGPGAAI
11]        pos= 130 score=0.001516 unnormalised=0.5365600000        GLSMAGSSA
12]        pos= 131 score=0.026878 unnormalised=9.5155600000        LSMAGSSAM
13]        pos= 133 score=0.014170 unnormalised=5.0165600000        MAGSSAMIL
14]        pos= 140 score=0.003450 unnormalised=1.2215600000        ILAAGPGPG
15]        pos= 141 score=0.009555 unnormalised=3.3825600000        LAAGPGPGT
16]        pos= 156 score=0.002838 unnormalised=1.0045600000        VQYSRAGPG
17]        pos= 158 score=0.015950 unnormalised=5.6465600000        YSRAGPGPG
18]        pos= 171 score=0.004476 unnormalised=1.5845600000        YGAMALAAF
19]        pos= 179 score=0.011941 unnormalised=4.2275600000        FHGPGPGMS
20]        pos= 191 score=0.018582 unnormalised=6.5785600000        YNINISLPS
21]        pos= 237 score=0.039120 unnormalised=13.8495600000        WSWLLSVLA
22]        pos= 241 score=0.007086 unnormalised=2.5085600000        LSVLAAGPG
23]        pos= 244 score=0.009134 unnormalised=3.2335600000        LAAGPGPGA
24]        pos= 266 score=0.008930 unnormalised=3.1615600000        YETAGPGPG
25]        pos= 275 score=0.030316 unnormalised=10.7325600000        YGYAAASAV
26]        pos= 277 score=0.035061 unnormalised=12.4125600000        YAAASAVAA
27]        pos= 287 score=0.007552 unnormalised=2.6735600000        LNPLGPGPG
28]        pos= 311 score=0.007860 unnormalised=2.7825600000        VAHGPGPGV
29]        pos= 323 score=0.009628 unnormalised=3.4085600000        VMPAAAAGS
30]        pos= 351 score=0.010580 unnormalised=3.7455600000        MAAGPGPGL
31]        pos= 359 score=0.009139 unnormalised=3.2355600000        LLLVLAFAG
32]        pos= 363 score=0.014619 unnormalised=5.1755600000        LAFAGGYAV
33]        pos= 365 score=0.017562 unnormalised=6.2175600000        FAGGYAVAA
34]        pos= 371 score=0.002936 unnormalised=1.0395600000        VAACAAYGS
35]        pos= 384 score=0.005933 unnormalised=2.1005600000        LSMAAYNAA
36]        pos= 389 score=0.025203 unnormalised=8.9225600000        YNAAGGHNA
37]        pos= 401 score=0.026867 unnormalised=9.5115600000        YTAAQAAVV
38]        pos= 409 score=0.011650 unnormalised=4.1245600000        VRAAYAMSG
39]        pos= 419 score=0.026822 unnormalised=9.4955600000        IVGAAAYRT
40]        pos= 431 score=0.011215 unnormalised=3.9705600000        INYRAAYAA
41]        pos= 433 score=0.022373 unnormalised=7.9205600000        YRAAYAAIG
42]        pos= 437 score=0.016082 unnormalised=5.6935600000        YAAIGGGVA
43]        pos= 449 score=0.027282 unnormalised=9.6585600000        YATATATAT
44]        pos= 461 score=0.002818 unnormalised=0.9975600000        YFLNLDVPL
45]        pos= 470 score=0.003572 unnormalised=1.2645600000        FVAAYQAET
46]        pos= 474 score=0.023669 unnormalised=8.3795600000        YQAETAVNT
```

```
47]        pos= 480 score=0.032914 unnormalised=11.6525600000        VNTLAAYAV
48]        pos= 488 score=0.017385 unnormalised=6.1545600000        VNGEPAPLA
49]        pos= 498 score=0.000233 unnormalised=0.0825600000        YGLSNATPR
50]        pos= 500 score=0.008334 unnormalised=2.9505600000        LSNATPREV
51]        pos= 522 score=0.002182 unnormalised=0.7725600000        AYNLPPPLA
52]        pos= 523 score=0.007651 unnormalised=2.7085600000        YNLPPPLAP
53]        pos= 542 score=0.008800 unnormalised=3.1155600000        FVRTSNIKF
54]        pos= 550 score=0.000137 unnormalised=0.0485600000        FQDAYNKKA
55]        pos= 563 score=0.000674 unnormalised=0.2385600000        ICTGGGNIM
56]        pos= 576 score=0.012190 unnormalised=4.3155600000        YVKKAGLSA
57]        pos= 588 score=0.011769 unnormalised=4.1665600000        LAHADSLAS
58]        pos= 594 score=0.010521 unnormalised=3.7245600000        LASSASKKI
59]        pos= 606 score=0.000527 unnormalised=0.1865600000        AEAMGNSRM
60]        pos= 616 score=0.014097 unnormalised=4.9905600000        YNQYRSKKA
61]        pos= 650 score=0.029994 unnormalised=10.6185600000        FEKLEPMAS
62]        pos= 653 score=0.001259 unnormalised=0.4455600000        LEPMASILD
63]        pos= 673 score=0.001459 unnormalised=0.5165600000        VLATQGRGA
64]        pos= 691 score=0.018983 unnormalised=6.7205600000        YHAMSSTHE
65]        pos=  -1 score=0.186902 unnormalised=66.1680000000        non-binding event

=================================================================

Allele: DRB1_1501
Pseudo sequence: RVTLGRDASW
Threshold: 3.125180
Max score: 28.118000


------------------------------
Antigen sequence file: /opt/lampp/htdocs/C-IMMSIM/Jobs/input/9107_20230207-091852_5_5fvJbmMTm4C6.FSA_1_001
------------------------------

IALTLAALPVAAMADVTLYGTIKAGVETSRSVAHNGAQAASVETGTGIVDLGSKIGFKGQEDLGNGLKAIWQVEQEAAAK
AGLFQRHGEGTKATVGEPVGPGPGTGSAVVGLSMAASSALTLGPGPGAAIGLSMAGSSAMILAAGPGPGTNIRQAGVQYS
RAGPGPGAQGGYGAMALAAFHGPGPGMSDPAYNINISLPSYYPDGPGPGALSAGVGAVAMSLTVGAGPGPGRRRSLRWSW
LLSVLAAGPGPGAELTAAQVRVAAAAYETAGPGPGYGYAAASAVAARLNPLGPGPGGTVGGAAPGGGAAAAVAHGPGPGV
APSVMPAAAAGSSATGPGPGTGAVLGGGGIAMAAGPGPGLLLVLAFAGGYAVAACAAYGSAVVGLSMAAYNAAGGHNAVA
AYTAAQAAVVRAAYAMSGDIVGAAAYRTADGINYRAAYAAIGGGVAVAAYATATATATLAAYFLNLDVPLFVAAYQAETA
VNTLAAYAVNGEPAPLAAYGLSNATPREVAAYQVTRNRIKKAAYNLPPPLAPDFKKAKFLEGFVRTSNIKFQDAYNKKAP
DDPICTGGGNIMAHVSYVKKAGLSASSGLAHADSLASSASKKIGQAAEAMGNSRMFYNQYRSKKALGQTVQASDSLTGAE
ETLNKKVNTLFEKLEPMASILDPGASKKAVGERVLATQGRGAWPVCGRKKAYHAMSSTHEANTMAMMARDEAAAKAPPHA
LSHHHHHH

Epitopes of protein 0 ------------
0]        pos=   9 score=0.015642 unnormalised=2.2588200000        VAAMADVTL
1]        pos=  15 score=0.030772 unnormalised=4.4438200000        VTLYGTIKA
2]        pos=  25 score=0.036035 unnormalised=5.2038200000        VETSRSVAH
3]        pos= 108 score=0.028023 unnormalised=4.0468200000        VVGLSMAAS
4]        pos= 113 score=0.032420 unnormalised=4.6818200000        MAASSALTL
5]        pos= 119 score=0.001799 unnormalised=0.2598200000        LTLGPGPGA
6]        pos= 133 score=0.011570 unnormalised=1.6708200000        MAGSSAMIL
7]        pos= 141 score=0.001065 unnormalised=0.1538200000        LAAGPGPGT
8]        pos= 156 score=0.019436 unnormalised=2.8068200000        VQYSRAGPG
9]        pos= 237 score=0.048658 unnormalised=7.0268200000        WSWLLSVLA
10]        pos= 311 score=0.004174 unnormalised=0.6028200000        VAHGPGPGV
11]        pos= 359 score=0.000580 unnormalised=0.0838200000        LLLVLAFAG
12]        pos= 362 score=0.011591 unnormalised=1.6738200000        VLAFAGGYA
13]        pos= 374 score=0.012512 unnormalised=1.8068200000        CAAYGSAVV
14]        pos= 419 score=0.004465 unnormalised=0.6448200000        IVGAAAYRT
15]        pos= 431 score=0.002409 unnormalised=0.3478200000        INYRAAYAA
16]        pos= 446 score=0.014125 unnormalised=2.0398200000        VAAYATATA
17]        pos= 471 score=0.014201 unnormalised=2.0508200000        VAAYQAETA
18]        pos= 480 score=0.047350 unnormalised=6.8378200000        VNTLAAYAV
19]        pos= 495 score=0.010220 unnormalised=1.4758200000        LAAYGLSNA
20]        pos= 570 score=0.057467 unnormalised=8.2988200000        IMAHVSYVK
```

```
21]        pos= 582 score=0.027787 unnormalised=4.0128200000        LSASSGLAH
22]        pos= 591 score=0.012747 unnormalised=1.8408200000        ADSLASSAS
23]        pos= 606 score=0.017400 unnormalised=2.5128200000        AEAMGNSRM
24]        pos= 612 score=0.037904 unnormalised=5.4738200000        SRMFYNQYR
25]        pos= 616 score=0.023764 unnormalised=3.4318200000        YNQYRSKKA
26]        pos= 653 score=0.017691 unnormalised=2.5548200000        LEPMASILD
27]        pos=  -1 score=0.458192 unnormalised=66.1680000000        non-binding event
```
